# Balancing selection via life-history trade-offs maintains an inversion polymorphism in a seaweed fly

**DOI:** 10.1038/s41467-020-14479-7

**Published:** 2020-02-03

**Authors:** Claire Mérot, Violaine Llaurens, Eric Normandeau, Louis Bernatchez, Maren Wellenreuther

**Affiliations:** 1 Département de biologie, Institut de Biologie Intégrative et des Systèmes (IBIS), Université Laval, 1030 Avenue de la Médecine, G1V 0A6 Quebec, Canada; 2 Institut de Systématique, Evolution et Biodiversité (UMR 7205 CNRS/MNHN/SU/EPHE), Museum National d’Histoire Naturelle, CP50, 57 rue Cuvier, 75005 Paris, France; 3 The New Zealand Institute for Plant & Food Research Ltd, PO Box 5114, Port Nelson, Nelson, 7043 New Zealand; 4 School of Biological Sciences, University of Auckland, 5 Symonds St, 1010 Auckland, New Zealand

**Keywords:** Evolutionary genetics, Experimental evolution, Structural variation, Entomology

## Abstract

How natural diversity is maintained is an evolutionary puzzle. Genetic variation can be eroded by drift and directional selection but some polymorphisms persist for long time periods, implicating a role for balancing selection. Here, we investigate the maintenance of a chromosomal inversion polymorphism in the seaweed fly *Coelopa frigida*. Using experimental evolution and quantifying fitness, we show that the inversion underlies a life-history trade-off, whereby each haplotype has opposing effects on larval survival and adult reproduction. Numerical simulations confirm that such antagonistic pleiotropy can maintain polymorphism. Our results also highlight the importance of sex-specific effects, dominance and environmental heterogeneity, whose interaction enhances the maintenance of polymorphism through antagonistic pleiotropy. Overall, our findings directly demonstrate how overdominance and sexual antagonism can emerge from a life-history trade-off, inviting reconsideration of antagonistic pleiotropy as a key part of multi-headed balancing selection processes that enable the persistence of genetic variation.

## Abstract (web summary)

Few studies empirically pinpoint how balanced polymorphisms are maintained. “Mérot et al”. identify an inversion polymorphism that is maintained in seaweed fly populations because of antagonistic pleiotropy that mediates a classic life history tradeoff between larval survival and adult reproduction.

## Introduction

The selective mechanisms involved in the maintenance of long-term polymorphism in the face of genetic drift often remain unknown. Early assessments of heritable diversity, as well as recent empirical genomic and theoretical studies, all emphasise the importance of balancing selection in promoting within-species diversity and maintaining intermediate allele frequencies^1–4^. The best-documented forms of balancing selection are overdominance, where heterozygotes benefit from a higher survival compared to homozygotes, and negative frequency-dependent selection, where allelic fitness decreases with increasing frequency, resulting in the protection of rare alleles. Balancing selection may also arise from the combination of opposing selection pressures, each favouring different alleles at polymorphic loci^5^, for example via spatially or temporally varying selection, where the fitness of the different alleles varies among habitats or seasons^6–8^, or sexually antagonistic selection, where allelic fitness varies between sexes^9–11^.

However, the mechanisms underlying balanced polymorphisms are still largely unidentified, partly because the fitness associated with different genotypes is affected by several interacting life-history factors. Individual fitness results from complex trait combinations of survival, longevity, reproductive success and fecundity, which can be under opposed selective pressures. For example, one allele can increase survival but weaken reproductive success, while the alternative allele can confer high fertility at the cost of decreasing survival, creating a life-history trade-off^12,13^. Such antagonistic pleiotropy increases genetic variation via the maintenance of intra-locus polymorphism^14,15^. Antagonistic pleiotropy has long been considered as a minor contributor to balancing selection because theoretical studies predicted it enables persistent polymorphism only for a limited range of parameters^16–18^. Nevertheless, recent models suggest that the role of antagonistic pleiotropy has been underestimated^19–22^ by showing that several factors, realistic in natural populations, can promote polymorphism persistence. These factors include trait-specific dominance (i.e. when the level of dominance varies between fitness components^22,23^), sex-specific selection (i.e. selection strength on each fitness component differs between sexes^19^) and spatially and temporally varying selection^20,21^.

These recent theoretical developments urge for a re-examination of the mechanisms allowing polymorphism maintenance in natural systems and to disentangle their effects on the different components of fitness. For example, detailed work on phenotypic variation in horn size in Soay sheep (*Ovis aries*) shows that antagonistic pleiotropy due to a life-history trade-off between survival and reproductive success at a single locus maintains polymorphism by causing an overall net heterozygote advantage^13^. Horn size is also under sex-specific selection and involves trait-specific dominance, two factors predicted to contribute to the maintenance of genetic variation. Similarly, in the yellow monkeyflower (*Mimulus guttatus*) variability in flower size is related to antagonistic pleiotropy due to a trade-off between viability and fecundity, with the persistence of the polymorphism being further enhanced by spatial and temporal environmental heterogeneity^20,24,25^. However, direct empirical evidence of antagonistic pleiotropy enabling long-term polymorphism remains scarce, in part because the different components of fitness are rarely estimated separately.

Here, we focus on the seaweed fly *Coelopa frigida*, whose natural populations are all polymorphic at a large chromosomal inversion on chromosome I, representing about 10% of the genome, 25 MB and containing one thousand genes^26–28^. Recombination within large inversions is suppressed between the different rearrangements. Consequently, inversions behave as a single locus, with alleles corresponding to the different haplotypic rearrangements^29–31^. In *C. frigida*, the two alleles, referred to as *α* and *β*, differ by more than 2.5% in coding regions and are observed at stable and intermediate frequencies in both European and North American *C. frigida* populations, suggesting that the haplotypic rearrangements diverged a long time ago and have been maintained ever since by balancing selection^26–28^. The widespread excess of *αβ* heterozygotes and the higher egg-to-adult survival of heterozygotes compared to *αα* and *ββ* homozygotes implicates that this polymorphism is partly maintained by overdominance, possibly due to deleterious alleles captured by the inversion^32,33^. Moreover, inversion frequencies correlate with environmental factors, suggesting that spatially varying selection combined with migration also contributes to maintain this polymorphism^26,27,34^. Previous work has also shown that the inversion strongly affects adult size, which is linked to female fertility and male reproductive success^35–37^, as well as development time^38^ which could modulate egg-to-adult survival. While the phenotypic effect on females is moderate, *αα* males can be up to three times larger than *ββ* males, but *αα* males can also take up twice as long to develop than *ββ* males^26,38^. This pattern suggests a trade-off between adult size and egg-to-adult development, which may result in a trade-off between fertility and survival. These findings make the inversion polymorphism in *C. frigida* a relevant empirical case to investigate the role of antagonistic pleiotropy in promoting polymorphism and to specifically test interactions with other mechanisms favouring the maintenance of variation, such as sex-specific effect, overdominance and spatially varying selection.

**Fig. 1:**
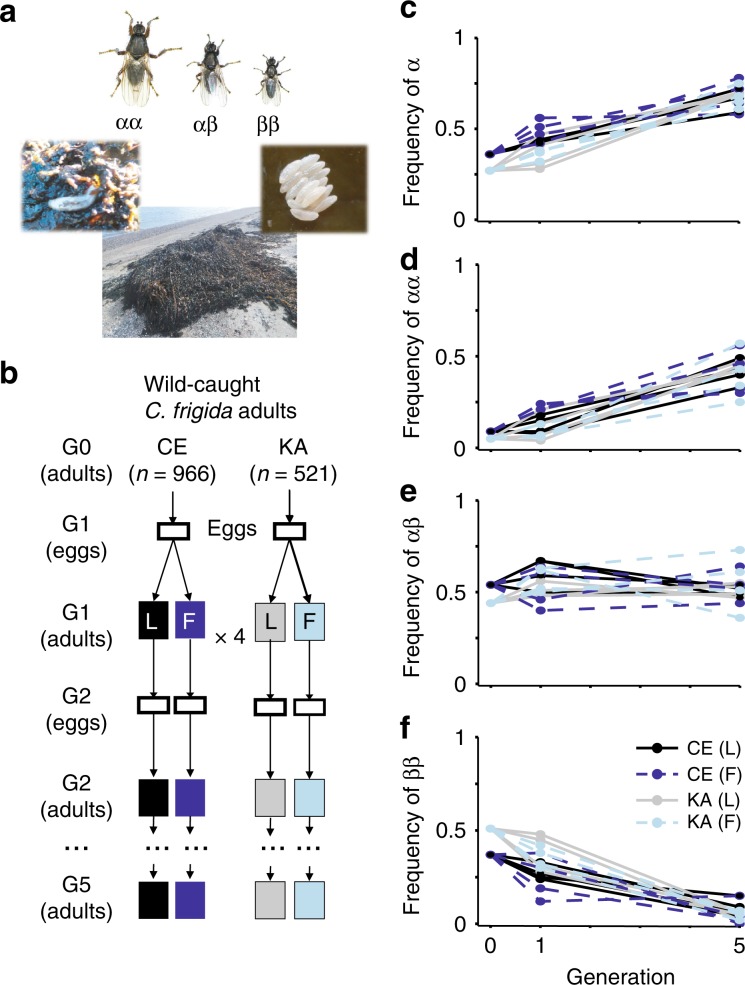
In vivo experimental evolution of *Coelopa frigida* and inversion dynamics. **a** *C. frigida* is a seaweed fly inhabiting seaweed wrackbeds that are accumulating and decomposing on the shoreline. Larva are exclusively restricted to this wrackbed substrate and adults are generally found crawling on or within the decomposing seaweed on which they lay eggs in clusters, although they can at times stray away from the wrackbed. Size variation in adult males is associated with the three genotypes of the inversion. Photos by C. Mérot & M. Wellenreuther. **b** Overview of the in-laboratory evolution experiment design. Starting with wild populations collected from two locations (CE & KA) in Québec (Canada), we raised 16 replicated experimental populations separately over five generations (denoted as G), either on a substrate dominated by Laminariaceae **L** or Fucaceae **F**. Eggs and adults were genotyped for a SNP marker associated with the inversion to infer genotype frequencies. **c**–**f** Evolution of the frequency of the inversion allele *α* and the proportion of each karyotypes between generation 0, 1 and 5. The same trend was observed in all 16 replicates for both KA and CE origins and on both substrates. Source data are provided as a Source Data file.

We combine experimental evolution and simulations to investigate the mechanisms of balancing selection underlying the maintenance of the inversion polymorphism in *C. frigida* (Fig. 1a) and to decipher the role of antagonistic pleiotropy in this process by estimating the effect of inversion alleles on different fitness components. First, we use experimental evolution (Fig. 1b) to follow the inversion frequencies over five generations and to estimate the relative fitness associated with each genotype. We determine the inversion genotypes in the eggs to specifically dissect the different components of fitness, such as egg-to-adult survival and reproductive bias. Second, realistic numerical simulations based on these experimentally estimated parameters allow quantifying the contribution of antagonistic pleiotropy in the maintenance of this inversion polymorphism. Both the experiments and simulations support that the life-history trade-off mediates balancing selection maintaining the inversion polymorphism, in interaction with other factors, namely sex-specific selection, trait-specific dominance and environment. Finally, we expand our simulation model to characterize how the combination of different selective mechanisms favours the persistence of polymorphism via antagonistic pleiotropy.

## Results

### Inversion dynamics during in vivo experimental evolution

The frequency of the *α* allele, originally at 27–36% in natural populations, increased significantly between generations 1 and 5 from 28–56% to 58–75% (glm *z* = 10.6, *p* < 0.001, Fig. 1c, Supplementary Tables 1 and 2). This was observed in all 16 replicates, with no significant effect of the substrate (glm *p* = 0.27, Supplementary Table 2). Initial differences in frequency between the two origins were lost at generation 5 (Fig. 1c–f, Supplementary Table 2). The increase in *α* frequency stemmed from a sharp and significant increase of *αα* homozygotes from 4–24% to 25–57% (glm *z* = 8.8, *p* < 0.001, Fig. 1d) and a reduction in *ββ* homozygotes from 12–48% to 0–15% between generation 1 and 5 (glm *z* = −7.9, *p* < 0.001, Fig. 1f). Proportions of *αβ* heterozygotes remained stable around 50% [36–73%] between generations 1 and 5 (glm *z* = −1.4, *p* = 0.16, Fig. 1e).

To disentangle the fitness components for the three genotypes and explore dominance effects, we genotyped the adults and the eggs at each generation for a subset of four replicates (Supplementary Table 1) and monitored frequency deviations due to biased survival or non-random reproduction. In combination with follow-up experiments and simulations, we evaluated four fitness parameters in the three genotypes: egg-to-adult survival, development time, female fecundity, and male reproductive success.

### The *β* allele confers a viability advantage to larvae

**Fig. 2:**
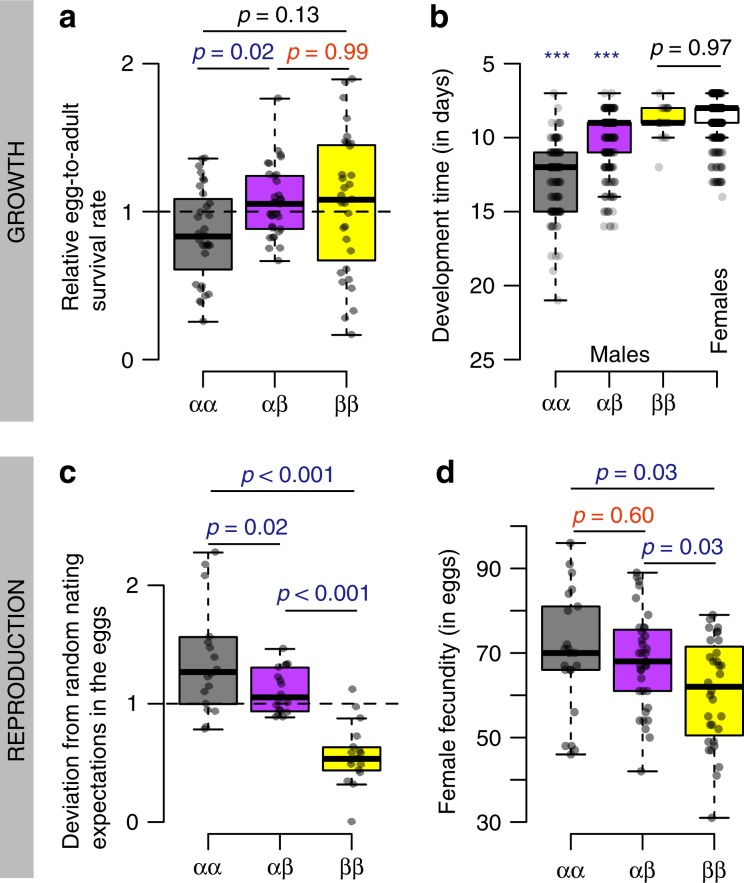
The pleiotropic and antagonistic effects of the inversion on different components of fitness. **a** Relative egg-to-adult survival rate per genotype, calculated as the deviation of each genotype’s proportion between adults and eggs (males and females were considered together). **b** Development time, measured as the number of days from the egg to the emerging adult for each combination of genotype and sex (the white box being developmental time for all females given that no significant difference was found between genotypes). **c** Deviation of genotypic proportions in the eggs relative to the proportions expected under random mating of the previous generation. **d** Female fecundity, measured as the number of eggs in the first clutch. Colours stands for the different inversion genotypes (*αα*, grey, *αβ* purple, *ββ*, yellow). Boxes indicate quartile, central line indicates the median and whiskers extend to 1.5 times the interquartile value. Overlapping points represent individual estimates per replicate. *P*-values, from post-hoc pairwise *t*-test, represent significant differences when indicated in blue and, when indicated in red, they represent non-significant differences between the heterozygotes and homozygote, suggesting dominance relationships between *α* and *β* alleles. Source data are provided as a Source Data file.

Egg-to-adult relative survival was significantly affected by genotype (lmm *df* = 2, *F* = 4.7, *p* *<* 0.01, Fig. 2a, Table 1, Supplementary Table 4, Supplementary Fig. 2). Survival of *αα* homozygotes was on average 21% lower than *αβ* heterozygote and l0% lower than *ββ* homozygote survival. Therefore, the increase of *αα* frequency during the experiments is a paradox given the lower viability of this genotype. Here, *ββ* and *αβ* relative survival rate did not differ significantly (mean difference 2%, *t*-test *p* = 0.99), suggesting a dominance effect of the *β* allele on egg-to-adult survival. This contrasts with the general finding of overdominance in European populations of *C. frigida*, where *αβ* heterozygote larvae survive better than both homozygotes^32,33,39^. Yet, the high *αβ* relative survival is known to be enhanced by increased competitive conditions^33^. Therefore, the performance of homozygotes in our data may be explained by the low-density conditions maintained in our experiment. We also calculated relative sex-specific survival rate of the three genotypes (Table 1). Although these estimates should be interpreted cautiously given their large variance, our data were consistent with previous estimates on *C. frigida* populations raised at low density^33^: overdominance of *αβ* was observed for male survival while *ββ* females tended to outperform *αβ* females (Table 1, Supplementary Fig. 1, Supplementary Table 3), suggesting that the effects of the inversion on viability as well as the dominance relationship differs between sexes.

**Table 1:** Fitness parameters depending on sex and genotype.

Sex	Fitness component	*αα*	*αβ*	*ββ*
Male	Egg-to-adult relative survival	*S*_*αα-m*_ 0.81 1−*s*_m_	*S*_*αβ-m*_ 1.0 1−*s*_m_*.H*_s_	*S*_*ββ-m*_ 0.88 1
	Relative reproductive success	*T*_*αα-m*_ 1.0 1	*T*_*αβ-m*_ (0.55) 1−*t*_m_*.H*_t_	*T*_*ββ-m*_ (0.1) 1−*t*_m_
Female	Egg-to-adult relative survival	*S*_*αα-f*_ 0.71 1−*s*_f_	*S*_*αβ-f*_ 0.90 1−*s*_f_*.H*_s_	*S*_*ββ-f*_ 1.0 1
	Relative reproductive success	*T*_*αα-f*_ 1.0 1	*T*_*αβ-f*_ 0.97 1−*t*_f_*.H*_t_	*T*_*ββ-f*_ 0.87 1−*t*_f_

Within each cell, the first line is the parameter name as defined in the “Methods” section, the second line is the numeric values inferred from the experiment and used by default in most simulations exploring a realistic set of parameters, the third line is a conceptualization of the parameter based on Zajitschek and Connallon^19^ used for the simulations exploring a theoretical space of parameters, with *s*_m_, *s*_f_ being the coefficients of selection for survival in males and females, and *t*_m_ and *t*_f_ being the coefficients of selection for reproduction in males and females. *H*_s_ is the coefficient of dominance for survival and *H*_t_ is the coefficient of dominance for reproduction.

The inversion also showed a sex-specific effect on the duration of the larval stage, with no significant difference among female genotypes (on average, *αα:* 9.0 days, *αβ:* 8.7 days, *ββ:* 9.0 days; glmm *p* = 0.57) but highly significant differences among males (glmm *p* < 0.001, Fig. 2b Supplementary Table 5, Supplementary Fig. 3). In our experimental conditions, (i.e. 25 °C and low-density) the development time was on average 50% longer for *αα* males (12.8 days), and 25% longer for *αβ* males (10.3 days) compared to *ββ* males (8.8 days). Such ordering between genotypes is consistent with previous studies, although absolute values are larger at higher density^38,40^ or lower temperature (pers. obs.). Therefore, the *α* allele is expected to confer an additional mortality risk in the natural environment for males by delaying sexual maturity, although this effect is challenging to quantify in the laboratory where the substrate is not limiting.

### The *α* allele confers a reproductive advantage

Egg genotype proportions significantly deviated from proportions expected under random mating, i.e. based on the Hardy–Weinberg proportions of the previous generation (combined probabilities of *Χ*² tests, *p* = 0.003). The deviation from random mating expectation in the eggs was significantly different between genotypes (lmm df = 2, *F* = 24.9, *p* < 0.001; Fig. 2c, Supplementary Table 6, Supplementary Fig. 4). It consistently corresponded to an excess of *αα* eggs (on average +43%), a slight excess of *αβ* eggs (+11%) and a strong deficit of *ββ* eggs (−44%). Those deviations mean that the *α* allele provided an important advantage for reproduction potentially underlying the increase of *α* frequency in our experiment.

The excess of the *α* allele in the eggs was partly explained by a higher fecundity of the females bearing the *α* allele. In a follow-up experiment on 90 females, *αα* and *αβ* females laid significantly more eggs than *ββ* females in their first clutch (*t*-test *p* = 0.03), with 15% more eggs on average (*αα*: 70 eggs, *αβ*: 68 eggs, *ββ*: 61 eggs—Fig. 2d), possibly due to the larger size associated with *α* also observed in females^35^. The number of eggs laid by *αβ* females and *αα* females did not differ significantly (*t*-test *p* = 0.60, Fig. 2d), suggesting that the advantage in female fecundity associated with the *α* allele may be dominant.

**Fig. 3:**
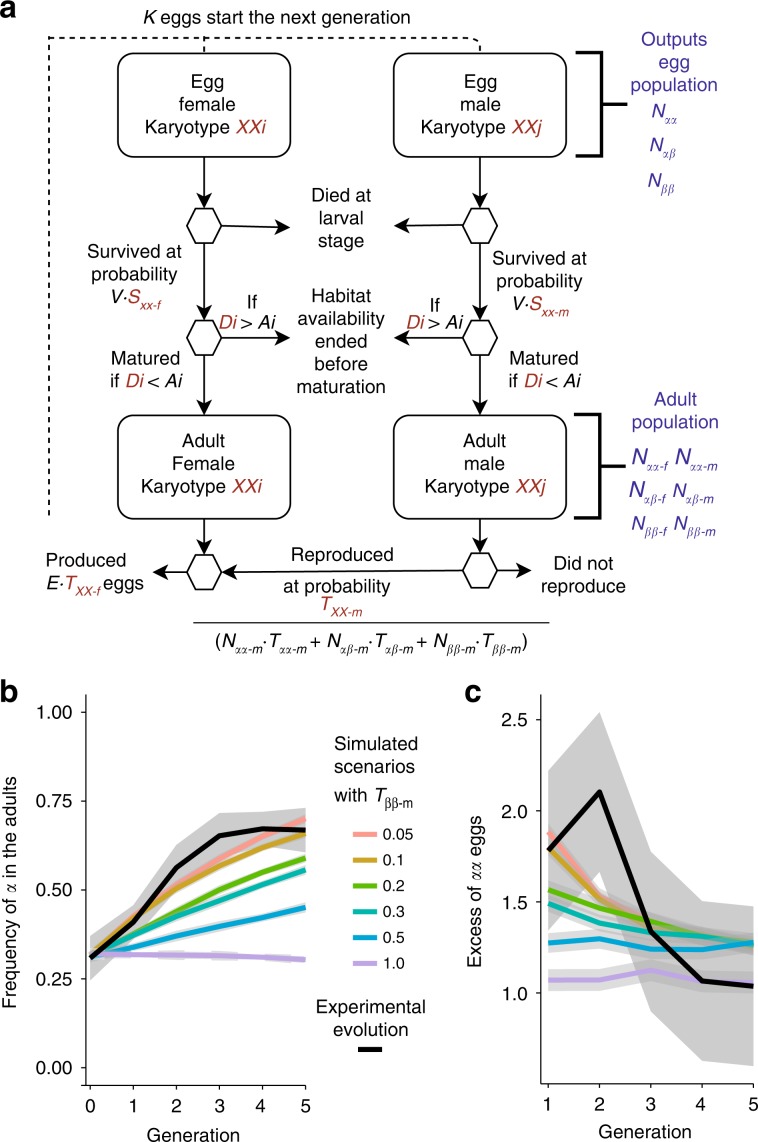
In silico evolution of *Coelopa frigida* and inversion dynamics over five generations. **a** Overview of the individual-based simulation model. Each egg is characterized by a genotype and a sex, inherited in a Mendelian fashion from its parents. Eggs survive through the larval stage at a probability determined by the product of *V* (global viability = 0.3) and *S*_*XX-s*_, the relative survival rate for each combination of sex and genotype (Table 1). The larva transitions into an adult only if its development time, an individual value (*D*_i_) drawn from a distribution determined by its genotype and sex, is shorter than its individual value of habitat availability (*A*_i_), drawn from a uniform distribution of habitat availability characterized by two parameters, mean duration (*A*_mean_) and variability (*A*_var_). For scenarios simulating laboratory experimental conditions, *A*_mean_ is set to a very large value (30 days). Adults go through a reproduction phase during which all females mate and lay a number of eggs determined by the product of the fertility parameter *E* (70 eggs) and *T*_*XX-f*_, the relative female reproductive success by genotype drawn from experimental estimate (Table 1). Male can reproduce several times. For each female, a random male is drawn from the pool of adult males at a probability *T*_*XX-m*_, the relative male reproductive success, determined by their genotype (Table 1), and based on the relative proportions of males in the population. The next generation starts with a subset of *K* eggs representing either the experimental procedure or a limited carrying capacity in nature. **b, c** Comparison of the evolution of *α* frequency and *αα* excess in the eggs over five generations in the experiment to simulated scenarios of in silico evolution based on experimental parameters. Data are smoothed using a loess method across four replicates per generation for experimental data and across 30 replicates per generation and per set of parameters for simulated data, and standard error of the mean bounds are represented by the grey shade. Source data are provided as a Source Data file.

The excess of *α* in the eggs may also result from non-random mating favouring *α* males because of their larger body size. Previous experiments in *C. frigida* with two males and one female demonstrated that the largest male sires a disproportionately higher number of the progeny^37^. The reasons are two-fold: smaller males lose male–male competition by being dislodged by larger males^41^; and, even in single-pair experiments, smaller males are more likely to be rejected by females during mating than larger males, with *ββ* males being 30% and 20% less attractive than *αα* and *αβ* males, respectively^36,39,42^. To estimate male reproductive success in our experiment, we built an individual-based model simulating the evolution of inversion frequencies in silico over five generations with all parameters drawn from the experiment except for the male relative reproductive success (Fig. 3a, Table 1, Supplementary Table 7). A scenario with equal male reproductive success across genotypes did not fit our experimental data (Fig. 3b), meaning that the difference in female fecundity is not sufficient to explain the observed rise in *α* frequency and the excess of *αα* in the eggs. The best model fit was achieved by a 10-fold higher mating success in *αα* compared to *ββ* males (*T*_*ββm*_ = 0.1, Fig. 3b, c, Supplementary Table 8), with co-dominant, intermediate values for *αβ*. Models involving dominance were explored but generally exhibited a lower model fit (Supplementary Table 8). Models including a negative frequency-dependent effect on mating success were also explored to account for a possible higher male–male competition between large-size males when the proportions of *αα* increased. Although those models adequately fit the experimental data, the global fit was not significantly better compared to simpler models (Supplementary Fig. 5, Supplementary Table 8).

### Modelling the impact of the trade-off on inversion dynamics

To explore how a life-history trade-off modulates inversion frequencies dynamics in nature and to test whether they can contribute to the maintenance of the polymorphism over time, we expanded the individual-based model to 200 generations and used a larger population size (*K* = 10,000). Our experiment provided realistic values for the different life-history parameters. Yet, the increase of *α* frequency to 50–75% in the experiment departs sharply from the lower *α* frequencies generally observed in natural populations (30–50%^26,34^). This means that there were key differences between wild and experimental conditions that the model needed to take into account. We identified three main differences: (i) our experimental boxes were likely less densely crowded than natural wrackbeds and density is known to affect relative egg-to-adult survival^33^, (ii) during the experiment, male–male competition was likely stronger than in nature because of restricted space and synchronized reproduction, which may have favoured large *αα* males over smaller *ββ* males, (iii) the experimental substrate was unlimited while, in nature, access to the resource is frequently disrupted by tides or storm-induced waves, which may prevent slow-developing males from reaching adulthood^43^. We thus explored how genotype proportions are affected by the following three parameters: (i) relative egg-to-adult survival, (ii) male relative reproductive success, and (iii) duration of habitat availability. We then estimated the probability of maintaining polymorphism vs. fixing one allele within both a realistic and theoretical (less constrained) parameter space.

**Fig. 4:**
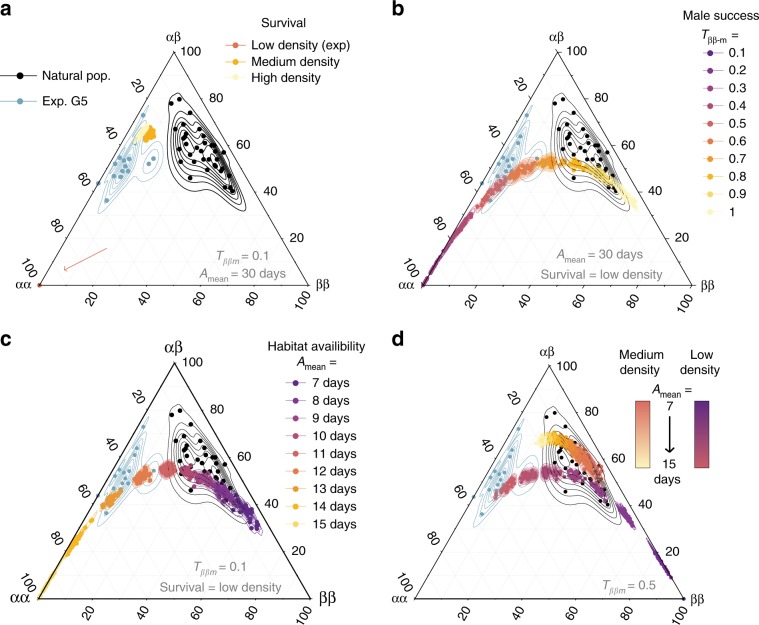
Inversion genotype proportions under simulated scenarios of in silico evolution. Ternary plots comparing the proportions of the three genotypes in natural populations^26, 27^, after the 5th generation of our laboratory experiment and at the equilibrium after 200 generations of simulations exploring the parameter space representative of the conditions encountered by *Coelopa frigida* in the wild. Simulated scenario vary **a** the effect of density, and the related relative survival rate, **b** the range of values for male relative reproductive success (*T*_*αα-m*_ = 1, *T*_*αβ-m*_ = 1/2(*T*_*αα-m*_ + *T*_*ββ-m*_), *T*_*ββ-m*_ = [0.1–1.0]), **c** the effect of a limited duration of the habitat availability (*A*_mean_ = [7–15 days], *A*_var_ = 2 days), and **d** the combined effects of density and environment for moderate differences of male relative reproductive success (*ββ* male reproductive success *T*_*ββ-m*_ = 0.5, i.e. two-fold lower than *αα* male reproductive success). Each point is either the proportion of genotypes for one natural population, one experimental replicate at G5 or the outcome of a single simulation. Lines draw a kernel density estimate for each group (natural populations, generation 5 of the experiment or each set of parameters). Source data are provided as a Source Data file.

The effect of density on egg-to-adult survival (i) could not account for frequency differences between the wild and the experiment (Fig. 4a). Yet, higher densities shifted the equilibrium proportions towards an excess of heterozygotes as observed in some natural populations (Fig. 4a, d).

Simulations reducing the relative reproductive success of *αα* males (ii) to about 2-fold (instead of 10-fold in our experiment) recovered an equilibrium of genotypic proportions close to values observed in the wild (Fig. 4b, d), confirming that the increase of *α* frequency in our experiments could be linked to a large reproductive advantage of males carrying the *α* allele, possibly intensified by male–male competition due to restricted experimental space and synchronised reproduction. In the wild, *ββ* males may also have easier access to females by reaching adulthood earlier than *αα* and *αβ* males. However, equal male reproductive success between the three genotypes led to higher *ββ* proportions than what is generally observed in nature and removing the *α*-female fecundity advantage led to the fixation of *β* allele. This suggests that the reproductive advantage conferred by the *α* allele in both males and females may also contribute to the persistence of *α*/*β* polymorphism in the wild.

**Fig. 5:**
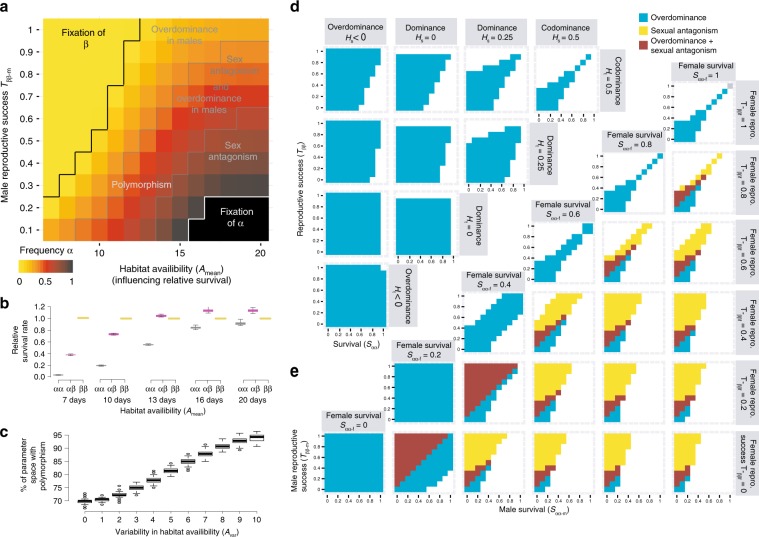
Conditions of maintenance of polymorphism through antagonistic pleiotropy. **a** Inversion frequencies at equilibrium and mechanisms of balancing selection emerging from the antagonistic pleiotropy, within the range of parameters explored for the *Coelopa frigida* wild populations. Female reproductive parameter values were based on experimental values, variability in the duration of the habitat (*A*_var_) was set to 2 days and relative survival rates correspond to low-density conditions. **b** Male relative survival according to the duration of habitat availability (*A*_mean_). Values are normalized relatively to a value of 1 for *ββ* males. Colours stands for the different inversion genotypes (*αα*, grey, *αβ* purple, *ββ*, yellow). Boxplots show the distribution of 30 replicated simulations, with boxes indicating quartile, central line indicating median and whisker expanding to 1.5 times the interquartile. **c** Variability in the duration of habitat availability (*A*_var_) further increases the portion of the parameter space leading to polymorphism persistence. The parameter space if defined by *T*_*ββ-m*_ = [0.1–1.0] and *A*_mean_ = [7–20 days], as in panel **a**. Boxplots show the distribution of 100 replicated simulations, with boxes indicating quartile, central line indicating median, whisker expanding to 1.5 times the interquartile and dots being outliers. **d** Outcome of simulations within a theoretical parameter space, without differences of parameters between sex nor any environmental effect. Overdominance in total fitness emerges as a result of antagonistic pleiotropy, even in the case of co-dominance for each fitness component (top right corner: *H*_s_ = 0.5, *H*_t_ = 0.5). Simulations in which dominance for survival (*H*_s_) and reproduction (*H*_t_) are independent show how a reversal of dominance and/or variation in the strength of dominance between components of fitness further expand the range of parameters leading to overdominance and the maintenance of polymorphism. **e** Outcome of simulations within a theoretical parameter space in which fitness parameters are independent between males and females (*S*_*αα-f*_ is the relative survival of *αα* females, *S*_*αα-m*_ is the relative survival of *αα* males, *T*_*ββ-f*_ is the relative reproductive success of *ββ* females, *T*_*ββ-m*_ is the relative reproductive success of *ββ* males), without dominance (*H*_s_ = *H*_t_ = 0.5) or without any environmental effect. Source data are provided as a Source Data file.

Finally, the duration of habitat availability (iii) appeared to be a prominent factor affecting genotypic proportions and explaining the difference in genotype frequencies between our experiment and wild populations (Fig. 4c). This was mediated by a different balance between survival and reproductive advantage. When the habitat availability was shorter, relative survival of *αα* males, and to a lesser extent of *αβ* males, was reduced in comparison to the faster-developing females (all three genotypes) and *ββ* males (Fig. 5b, Supplementary Fig. 6). It is noteworthy that variation in the duration of habitat availability tended to cover the natural variability in genotypic proportions (Fig. 4c, d), suggesting that spatio-temporal heterogeneity in habitat availability could explain the variable balance of genotypes observed in nature.

### Polymorphism maintenance through antagonistic pleiotropy

Taken together, the antagonistic pleiotropy among inversion-associated fitness components, as revealed by our experimental evolution approach, can explain the maintenance of the inversion polymorphism in nature, albeit considering a different tuning of the trade-off between survival and reproduction. Simulations exploring these different factors simultaneously showed that the polymorphism is maintained for a wide parameter space of male reproductive success and environmental conditions (Fig. 5a, Supplementary Fig. 7), even at low density conditions, where heterozygote survival advantage is very weak (Fig. 5b, Supplementary Fig. 6). Remarkably, when the duration of the habitat availability was more variable, the range of conditions maintaining polymorphism was considerably expanded (Fig. 5c). Also, the frequency of the inversion was buffered around intermediate values (Supplementary Fig. 8), which suggests that spatially varying selection resulting from environmental heterogeneity strongly favours the coexistence of alternative life-history strategies and thus the underlying genetic polymorphism. As expected, in models accounting for strong overdominance in survival (medium density conditions, Supplementary Fig. 9) or negative frequency-dependant effect on male success (Supplementary Fig. 10), most combinations of parameter values led to a protected polymorphism.

Estimating total fitness, defined as the combination of egg-to-adult survival and reproductive success for each genotype and sex, showed that antagonistic pleiotropy translates into two mechanisms of balancing selection: overdominance and sexual antagonism (Fig. 5a). Overdominance naturally emerges from antagonistic pleiotropy, even in the absence of dominance for any given trait, particularly when selection is strong (Fig. 5d). In the case of *C. frigida*, heterozygotes benefit from the combination of a survival advantage associated with *β* and increased reproductive success associated with *α*. Variation in the strength and direction of dominance between fitness components, as observed in our experiment, with for instance overdominance in *αβ* male survival and dominance of *β* allele in female larval survival (Fig. 2), considerably expanded the parameter space leading to overdominance and polymorphism maintenance (Fig. 5d). Sexual antagonism emerges from the sex-specific effects of each allele on survival and reproduction (Fig. 5e), and expands the range of conditions allowing polymorphism.

## Discussion

Results from the experimental evolution trial coupled with simulations show that the inversion dynamics in *C. frigida* can be largely explained by an antagonistic relationship between viability and fecundity. Individual-based simulations revealed that this antagonism is probably stronger in natural conditions and contributes to the widespread maintenance of inversion polymorphism in the wild. Both empirical results and simulations emphasize the importance of accounting for the reproductive phase and sexual selection. In fact, when considering viability alone, the persistence of the *α* haplotype, and its increase in our experiment, represents a paradox that is only resolved by considering female fecundity and the reproductive bias favouring larger males^37,42^. The antagonistic pleiotropy between growth and reproduction observed in *C. frigida* at the inversion also corroborates the evidence for trade-offs mediated by body size^44–46^. Such trade-offs frequently emerge because a bigger size either requires prolonged development time or a faster growth rate, both mechanisms associated with a lower likelihood to reach the reproductive stage. For instance, artificial selection for large body size in the yellow dung fly (*Scathophaga stercoraria*) increases juvenile mortality, particularly in stressful environments, as well as development time, leading to mortality before reproduction due to winter frost^47^. For a wide range of species, environmental factors impose such limits to development time. For example, in snow voles (*Chionomys nivalis*) the benefits of higher body size are counteracted by the need to reach adult size before the first snowfall^48,49^. Similarly, in many annual plants, delayed flowering allows investment in vegetative growth and a subsequently higher number of seeds, but is constrained by the duration of the reproductive window^20,24,50^. In *C. frigida*, this effect is relevant given the instability of the wrackbed habitat due to tide and wind induced waves (here modelled as limited habitat availability). By disproportionately increasing mortality of the genotypes with larger reproductive success, the effect of the environment is expected to exacerbate the trade-offs reported during the experimental trials.

When life-history trade-offs are affected by external factors, environmental heterogeneity in space and time strongly contributes to the maintenance of variation by locally favouring different life-history strategies. For instance, in the yellow monkeyflower *Mimulus* spp., spatial heterogeneity in wetness and seasonal variation alternatively favours alleles determining early-flowering/low fecundity or late-flowering/high fecundity^20,24,25^. In *C. frigida*, wrackbed stability varies between locations and across seasons depending on the exposure of the beach to tidal and storm-induced waves. Such heterogeneity is expected to generate fluctuating selection regimes between a slow-development/high fertility strategy (*αα*) or a fast-development/low fertility strategy (*ββ*), leading to variations in inversion frequency, and enhancing the maintenance of polymorphism. This hypothesis, originally proposed by Day, Butlin et al.^27,34^, is supported by field observations reporting geographic variation in genotypic proportions coinciding with tidal pattern in Scandinavia^27^ and temporal variation in genotypic proportions in Great Britain, with the *α* allele increasing in frequency in summer when the wrackbed was less frequently disturbed by storms^34^. Geographic variation in genotypic proportions in natural populations of *C. frigida* are also associated with environmental variations, such as air temperature, depth and temperature of the wrackbed and substrate composition^26,27,34^. Although those factors may correlate with the duration of the wrackbed stability, they are also known to modulate the genotype–phenotype relationships and therefore the associated fitness. Controlled experiments in *C. frigida* show that substrate composition, temperature and density affect the relationship between genotype and survival, development time, body weight and body size (G × E effect)^33,38,51,52^. In nature, this translates to geographic variation in male and female sizes, as well as variation in size difference among genotypes and between the sexes^26,36^. This is non-trivial because adult size, and size difference between sexes or between competitors may modify the reproductive advantage associated with the *α* allele. For instance, experiments showed that a larger size difference between two competitor males relates to a higher success of the larger male^37^. In other words, local environmental conditions are expected to affect genotype-associated fitness values both for viability and reproductive success. This may enhance the effect of spatial or temporal heterogeneity by changing the slope of the trade-off between survival and fertility in *C. frigida*. This feature is likely more general and many life-history trade-offs are probably modulated by environmental effects, since genetic correlations are known to vary in direction and strength between environments^53^. Spatio-temporal environmental heterogeneity thus appears to have a bivalent effect, by causing not only fluctuations in selection but also in the intensity of the trade-off. Both of these aspects interact and favour the maintenance of genetic variation.

Polymorphism generated by antagonistic pleiotropy is also predicted to be enhanced by different trade-off intensities for each sex^19^. Sex-specific selection on different fitness components frequently occurs in nature, since optimal age/size at maturity or the physiological state to achieve high fertility often differs between sexes^9,54^. *C. frigida* provides an empirical case of antagonistic pleiotropy whereby the strength of selection, but not the direction, differs between sexes for each fitness component. Differences between genotypes in size, development time, fertility and survival are stronger for males than for females^33,35,36,52^. Within a range of realistic parameters, sex-specific effects, even without antagonism for each fitness components, results in sexual antagonism for total fitness, therefore strongly protecting polymorphism^19^. Moreover, even without sexual antagonism in total fitness, our simulations suggest that sex-specific selection varying between components of fitness delays the fixation of alleles (Supplementary Fig. 11). While this mechanism cannot on its own protect long-term polymorphism, the slower rate of allelic fixation might allow additional factors, such as environmental heterogeneity, to prevent allele fixation^22,55^.

Overdominance emerges as a by-product of antagonistic pleiotropy, since the total fitness of heterozygotes is generally higher than both the fitness of homozygotes: *αβ* heterozygotes reach the highest fitness by combining a reproductive advantage due to large size (larger than *ββ*) and a survival advantage in natural conditions due to a shorter development time (shorter than *αα*). The parameter space leading to emergent overdominance is even more extended when dominance varies between fitness components, especially in the case of dominance reversal, that is when advantageous alleles for each component of fitness are dominant^18,23^. Since the reversal of dominance between fitness components has only been explored theoretically so far, it is still unknown how common it is in nature and what genetic architecture may underlie it. Our data still suggests that, in *C. frigida*, dominance at the inversion locus does vary in strength and direction between survival and fertility, as well as between sexes. These findings are in line with recent studies in other species showing that dominance can vary depending on the sex^9,10^, and that multivariate trait often involve the combination of trait-specific dominance^56,57^. Overdominance may also emerge from the genetic architecture of the trait under antagonistic pleiotropy: inversions are frequently associated with deleterious effects, either because the breakpoints disrupt a gene, or because they contain recessive deleterious mutations which can only be purged by recombination and purifying selection when they occur at the homozygous state^29,58–60^. The generally higher egg-to-adult viability of heterozygotes, which is enhanced in high-density conditions^33^, led to the the hypothesis that the two haplotypic rearrangements of *C. frigida* may each contain different clusters of deleterious mutations, as suggested by intra-population and inter-population crosses^32^. Such a linked genetic load may explain why the rarest *αα* genotype exhibits the highest deficit compared to Hardy–Weinberg expectations in nature^26^ and why the *αα* genotype has the lowest viability in laboratory conditions. Since *α* is less frequently found at the homozygous state than the *β* allele in nature, the purging of deleterious mutations linked to the *α* allele might be more limited than for the *β* allele. Such overdominance emerging from genetic load was thought to be the major mechanism underlying the maintenance of polymorphism in *C. frigida*^32,33^. Yet, depending on the conditions of growth and on sex, the viability advantage of heterozygotes is not necessarily strong, and a heterozygote excess can also emerge in the eggs, emphasizing the role of non-random mating. Overdominance thus appears linked to two mechanisms, antagonistic pleiotropy and associated genetic load, which possibly enhance each other. Overdominance emerging from antagonistic pleiotropy may generate an excess of heterozygotes, which in turn limit the purging of the different recessive deleterious mutations associated with the two haplotypes. Such a sheltering of the genetic load associated with the inversions may in turn reinforce overdominance.

Inversions are frequently reported as polymorphic and maintained over long evolutionary timescales by balancing selection^30,31,61,62^. While one of the reasons could be the genetic load linked to the lack of recombination, we argue that antagonistic pleiotropy may also be a more important feature of the inversion systems than previously acknowledged. In fact, the particular architecture of inversions leads them to behave as a single-locus because of the stark reduction of recombination between rearrangements, but they are composed of dozens to hundreds of genes, sometimes interacting in a so-called “supergene” complex^29,63,64^, where combinations of alleles at different genes lead to highly differentiated phenotypes. Inversion haplotypic rearrangements can thus have large, pleiotropic effects on complex phenotypes, which will be under various selective pressures, possibly in opposing direction^30^. For example, in the longwing butterfly *Heliconius numata* an inversion polymorphism determining wing colour pattern is under opposing pleiotropic selection, with survival under positive frequency-dependent selection and reproduction under negative frequency-dependent selection^65^. In the ruff *Calidris pugnax*, a polymorphic inversion that determines male reproductive morphs carries inverted alleles with lethal effect on survival but positive effects on testis size, indicating a possibly higher reproductive success, which is also under frequency-dependent selection^66^. In the rainbow trout *Oncorhynchus mykiss*, alternate reproductive tactics are determined by a large polymorphic inversion, which involves a trade-off between reproductive output and longevity^67^. The accumulating evidence from the literature combined with our results on *C. frigida* thus indicate that contrasted effects of inversions on different fitness components may represent a non-negligible process involved in the maintenance of inversion polymorphism. Clearly, antagonistic pleiotropy and the effect of a wide spectrum of fitness traits on pleiotropic interactions must be considered more carefully when studying the evolutionary importance of structural genomic variants, which are currently increasingly documented to underlie complex phenotypes^30,31,61^.

## Methods

### Experimental evolution in vivo

Wild *C. frigida* adults were collected at two locations along the Canadian Atlantic coast, in May 2017 at Cap Espoir, Québec (CE: 48.43087, −64.32778) and in June 2017 at Kamouraska, Québec (KA: 47.56294, −69.87375). The two populations (KA and CE) were kept separated and represented two distinct experimental lines. Wild-caught flies (KA: 303 females, 218 males, CE: 396 females, 570 males) constituted the generation 0 of the experimental evolution and were used to initiate 20 replicates of experimental evolution (2 populations × 2 substrates × 5 replicates).

To disentangle the fitness components, the generations were non-overlapping and we explicitly separated the growing phase from the reproductive phase. For reproduction, the pool of adults was left overnight at 25 °C on a layer of seaweeds, either Laminariaceae or Fucaceae. After 16–20 h, adults were preserved in ethanol. Eggs were collected by flooding the seaweed substrate with 3% salt water in each box. Egg density in the salt water solution was counted over a grid in three subsets of 3 mL each and a volume corresponding to an estimated 1000 eggs was filtered over a dark piece of linen. This subset of 1000 eggs was transferred to a controlled mass of seaweeds to start the growing phase of the next generation, and a subset of the remaining eggs was preserved in ethanol or RNAlater for subsequent genotyping. Upon emergence, adults were collected every day by aspiration and kept in conditions favouring high survival but without reproduction (5 °C, dark, with a solution of Mannitol 0.5%). When all adults emerged, we initiated the following generation with the same procedure, allowing free mating between all adults emerging from one replicate.

Each replicate was kept isolated and maintained for five generations under semi-natural conditions, controlling for density, temperature (25 °C), humidity (50%), light duration (12 h) and substrate. Raising boxes were identical plastic boxes measuring 12 × 8 × 18 cm closed with a hermetic lid perforated with four holes filled with sponge to allow air exchange but preventing adult flies to escape. Stones and sand were put in the bottom of the box to facilitate drainage. Half of the replicates were kept on 90% Laminariaceae/10% Fucaceae and the other half on 90% Fucaceae/10% Laminariaceae, the two main substrates on which *C. frigida* flies are naturally found in this region (Fig. 1b). Seaweeds were collected (either attached to the substrate or floating in the sea) in Métis sur Mer, Québec (48.66408, −68.07221) repeatedly over the course of the experiment. Seaweed were transported to Laval University and frozen for subsequent use as substrate in the experiment. Freezing allows a good conservation of the seaweed and destroys any eggs, parasites or larvae. The 1000 eggs started on 500 g unfrozen and cut seaweed substrate with subsequent addition of 200 g of seaweeds after ~6 days. This procedure ensured a controlled larval density between replicates.

Only 16 replicates (8 replicates per population) were kept until generation 5 due to stochastic crashes in population size experienced by some replicates because, to avoid founder effects, we allowed reproduction and the initiation of a subsequent generation only for those replicates in which more than 100 adults emerged per generation.

The frequency of the inversion and the proportions of the three inversion genotypes (*αα, αβ, ββ*) were estimated by genotyping adults (40–95 per replicate) and eggs (28–51 per replicate), using a diagnostic SNP assay^26^ after having extracted genomic DNA for each individual adult/egg with a lysis procedure. Variation in allele or genotype proportions between generations 1 and 5 was analysed with a generalized linear-mixed model for binomial data in the R package *lme4*^68^ and *lmerTest*^69^ taking into account the identity of the replicate as a random factor and substrate or population as covariates.

### Estimates of fitness

Relative survival rate of each genotype (and sex) was calculated by comparing the genotypic proportions in adults to the genotypic proportions observed in the eggs. The ratio of these values is expected to be 1 if all genotypes survive equally. Survival rate was calculated in 32 replicates (16 replicates at generation 1, 4 replicates at generation 2, 3, 4 and 5). Similarly, we calculated the deviation from random mating in the eggs as the ratio of genotypic proportions observed in the eggs over the expected proportions under random mating, i.e. Hardy–Weinberg proportions of the previous generation. This ratio is expected to be around 1 if mating occurs randomly and if all genotypes reproduce equally. Deviation from random mating in the eggs was estimated in 18 replicates (2 populations at generation 1, and 4 replicates at generations 2, 3, 4 and 5). Differences between genotypes and between sexes in survival rate or in the deviation from random mating in the eggs were tested with a linear mixed model, taking into account the identity of the replicate as random factor, and a paired post-hoc *t*-test corrected following Benjamini and Hochberg^70^.

Development time was measured at generation 5 on 757 adults as the number of days between the day of oviposition and adult emergence. Differences in development time between genotypes and sex were tested with a generalized linear-mixed model, based on a Poisson distribution, taking into account the identity of the replicate as a random factor. Each factor and interaction was added sequentially and nested models were built with the R package lme4^68^ and compared to each other with a *χ*²-test to determine whether the additional factor significantly improved the explicative power of the model.

Female fecundity was assessed in a follow-up experiment on a laboratory population established from eggs laid by wild-caught females from Kamouraska, Québec, raised under the same conditions as described for the previous experiment (25 °C, substrate of 90% Laminariaceae–10% Fucaceae, low density). After 7 days, pupae were collected every day and kept isolated in a 2 mL tube with cotton soaked in 0.05% Mannitol. Upon emergence, adults were kept for a few days at 5 °C in the dark. A total of 161 pairs of one male and one female were formed. On the day of the experiment, each pair was transferred into a small box (5 × 5 × 5 cm) with a mix of Laminariaceae and Fucaceae, and left overnight (for 16 h) at 25 °C (identical conditions as used in the previous experiment for mating and egg laying). After 16 h, if a clutch of eggs was observed, it was collected and preserved on a dark-linen in a 1.5 mL tube of RNAlater, and the parents were preserved in ethanol. If no eggs were observed, the pair was kept in the same experimental box and the eggs were checked again after an extra 8 h. We did this to maximize the number of females for which we could count eggs (eggs after 16 h: 69 females, eggs after 24 h: 120 females). We genotyped 90 females for the inversion using the method described above and counted their eggs under a binocular magnifier (Zeiss Stemi 2000C). Variation in the number of eggs per female in relation to female genotype (*αα, αβ, ββ*) and time of laying (16 h/24 h) was analysed with a linear model and post hoc pairwise *t*-test (adjusted following Benjamini and Hochberg^70^). Whether eggs were laid after 16 or 24 h did not significantly affect the number of eggs per female (*F*_1,88_ = 0.09, *p* = 0.76), or the interaction with genotype (*F*_2,84_ = 1.96, *p* = 0.15), and we therefore pooled the data for all subsequent analyses to derive female fitness parameters in the model.

All statistical analyses were performed using the software R 3.5.0^71^.

### Simulating evolution in silico

To evaluate how fitness differences between genotypes based on a different investment in the trade-off between survival and reproduction modulates the evolution of the inversion frequency, we developed an individual-based model inspired by the results of the experimental evolution trials and by the biological characteristics of *C. frigida* (Fig. 3a).

The model is fully described in Supplementary Methods following the ODD protocol^72^ and the basic principles of the model are as follows: generations are non-overlapping, the time step is one generation and each generation proceeds in two phases, growth and reproduction. Growth phase: Each egg goes through a growing period, during which its survival is determined by chance following a binomial law with a survival probability determined by the product between global egg-to-adult survival rate (30%) and relative genotype-sex-specific survival (*S*_*XX-s*_) depending on its sex (*s* = f or m) and genotype (*XX* = *αα* or *αβ* or *ββ*). The surviving larva then matures into an adult if its development time is shorter than the duration of its habitat. Individual development time (*D*_i_), is calculated for each individual larva as the cubic root of three values randomly drawn from a uniform distribution whose mean and range depends on sex and genotype (means are the experimental estimates reported in the result section for each genotype and sex, and range is determined with a variation coefficient of 0.5). We chose this distribution as the one fitting best the measured experimental data of the development time. The duration of habitat availability is randomly drawn for each individual larva from a uniform distribution determined by two parameters: mean duration (*A*_mean_) and variability (*A*_var_). Mean duration is shared by all individuals of a given simulation, and thus takes into account the non-independence in the availability of the habitat, i.e. the fact that when the wrackbed is removed by a high tide or a storm, all individuals are affected at the same time. Variability represents individual heterogeneity in the total duration before the wrackbed is removed, which depends for instance, at which date a given egg was laid or whether all patches of wrackbed are deposited or removed at the same time. Reproduction phase: All females reproduce once and lay a number of eggs determined by the product between the number of eggs laid by a female (70 eggs) and genotype-specific female fertility (*T*_*XX-f*_). For each female, a male partner is randomly drawn from the pool of reproductive males. This pool of reproductive males is generated at each reproductive step based on the distribution of adult males {*N*_*αα-m*_*; N*_*αβ-m*_*; N*_*ββ-m*_}, corrected by genotype-specific male reproductive success (*T*_*XX-m*_). Note that this procedure allows to account for the fact that each male can mate several times. Additional models taking into account a frequency-dependant effect are presented in Supplementary Information. Egg genotype is determined by Mendelian inheritance from parental genotypes, by randomly drawing one allele from the mother, and one allele from the father, and egg sex is determined by chance with no bias (sex-ratio = 0.5). A subset of *K* eggs initiates the next generation, mirroring the census made in the experiment or a limited carrying capacity in the wild. At each generation, we record the proportions of the three genotypes in the eggs and the adults. The model was implemented in Rust 1.32.0 (9fda7c223 2019-01-16).

The model outcomes were analysed at three levels.

(i) First, we ran the model for five generations with parameters drawn from the experiment (Table 1, Supplementary Table 7) and no limited duration of habitat availability (*A*_mean_ = 30 days) while varying the relative male reproductive success (30 replicates per set of parameters). The fit of each simulation to empirical data was quantified by computing the normalized root‐mean‐squared error (nRMSE) for each genotypic proportion from generation 1 to 5. The average nRMSE over the six variables (*αα*/*αβ*/*ββ* proportions in the eggs and the adults), and over the 30 replicates, was taken as an index of fit, with the best predicting scenarios having the smallest values. Difference of mean nRMSE between the best scenarios was tested with a *t*-test based on the 30 replicates, corrected following^70^.
(ii) Second, to simulate evolution in a natural population, we then ran the model with larger *K* (10,000) for 200 generations, with 30 replicates per set of parameters, and included variation in habitat availability (Supplementary Table 7). The equilibrium in genotypic proportions was compared to the frequencies observed in nature and we explored the combination of parameters possibly influencing the frequency of the inversion. The proportion of the three genotypes under each scenario was visualized in ternary plots built with the R package *ggtern*^73^. We tested the role of density by exploring several sets of survival values based on previous laboratory studies^33^ (Supplementary Table 3). For relative male reproductive success, the full range of parameters was explored because this parameter could not be estimated empirically and possibly varies between populations with natural variation in adult size^26,51^. Habitat availability was set between 7 and 20 days, i.e. the range of development time found in our experimental conditions, to estimate the interplay between those two factors. Yet, in natural conditions, both the range of wrackbed availability and development time are expected to be wider. Development time varies with density and temperature, although the ordering of emergence between the three male genotypes remains unchanged. Wrackbeds are expected to be removed cyclically by spring tides, so the actual availability would be slightly less than 14 or 28 days, but they are sometimes observed to last shorter because of storms^43^. Finally, for the parameters that are more likely to vary in natural populations (male relative success, duration of habitat availability, density, variability in the duration of habitat availability), we explored which combinations of realistic parameters maintained polymorphism after 200 generations, what was the mean frequency of the inversion at equilibrium after 100 replicates, which portion of the parameter space lead to polymorphism and whether overdominance or sexual antagonism emerged for total fitness.
(iii) Third, the backbone of the model was used to theoretically explore the range of conditions under which antagonistic pleiotropy could maintain polymorphism at evolutionary time-scales when combined with sex-specific effects and dominance, (*K* = 10,000, 500 generations, 100 replicates per set of parameters, initial proportions were set to Hardy–Weinberg proportions, with the frequency of *α* being 0.5).These simulations explored the whole theoretical parameter range for survival and reproduction (Table 1, Supplementary Table 7), with either various scenarios of dominance, coded by the parameters *H*_s_/*H*_t_, or sex-specific effects with independent values for *s*_m_/*t*_m_ and *s*_f_/*t*_f_ ranging between 0 and 1. We surveyed the proportions of the simulations that led to maintenance of polymorphism vs. the simulations in which one of the genotypes got fixed, as well as the emerging mechanism at the level of total fitness (overdominance in one/both sex, sexual antagonism).

Total fitness (*W*_*XX-s*_) was calculated as the product of relative survival rate and reproductive success, for each genotype or allele as

1 $$ W_{xx - s} = S_{xx - s} \cdot T_{xx - s}$$

2 $$ w_{\alpha - s} = w_{\alpha a - s} \, + \, \frac{1}{2} \, \cdot \, w_{\alpha \beta - s} $$

3 $$ w_{\beta - s} = w_{\beta \beta - s} \, + \, \frac{1}{2} \, \cdot \, w_{\alpha \beta - s} $$

Overdominance for total fitness corresponded to cases in which

4 $$ w_{a\beta - s} \, > \, w_{\alpha \alpha - s}\,\, \& \,\, w_{a\beta - s} \, > \, w_{\beta \beta - s} $$

Sexual antagonism for total fitness emerged in cases under which

5 $$ w_{a - m} \, > \, w_{\beta - m}\,\, \& \,\, w_{a - f} \, < \, w_{\beta - f} $$

or

6 $$ w_{a - m} \, < \, w_{\beta - m}\,\, \& \,\, w_{a - f} \, > \, w_{\beta - f} $$

### Reporting summary

Further information on research design is available in the Nature Research Reporting Summary linked to this article.

## Supplementary information

Supplementary Information

Peer Review File

Reporting Summary

## Data Availability

The authors declare that all data supporting the findings of this study are available within the paper and its supplementary information files. The experimental and simulated data underlying all figures and all supplementary figures are provided as a Source Data file.

## Source data

Source Data

## Code availability

Simulation code and parameters files are available at https://github.com/enormandeau/coelopa_fastsim.
