# Supplementary material for: Balancing selection via life-history trade-offs maintains an inversion polymorphism in a seaweed fly
Source: Nat Commun. 2020 Feb 3;11:670. doi: 10.1038/s41467-020-14479-7 (PMC6997199; doi:10.1038/s41467-020-14479-7)
Supplement: Supplementary file 1 — Supplementary Information [file 41467_2020_14479_MOESM1_ESM.pdf]

Supplementary Information:

Balancing selection via life-history trade-offs maintains an inversion polymorphism in a seaweed fly

Claire Mérot[1*], Violaine Llaurens[2], Eric Normandeau[1], Louis Bernatchez[1#], Maren Wellenreuther[3,4#]

[1]Département de biologie, Institut de Biologie Intégrative et des Systèmes (IBIS), Université Laval, Canada

[2]ISYEB (UMR 7205 CNRS/MNHN/SU/EPHE), Museum National d'Histoire Naturelle, Paris, France

[3]The New Zealand Institute for Plant & Food Research Ltd, Nelson, New Zealand

[4]School of Biological Sciences, University of Auckland, Auckland, New Zealand

*claire.merot@gmail.com

# These authors jointly supervised this work

Supplementary methods.................... 3
Simulation model describe following the ODD procedure.................... 3

Supplementary Tables and Figures.................... 6
Evolution of genotypic proportions in the experimental evolution.................... 6
Supplementary Table 1: Number of samples genotyped and proportions of the three genotypes in the experiment. 6
Supplementary Table 2: Analysis of genotypic proportions....................7
Egg-to adult relative survival in the experimental evolution.................... 7
Supplementary Table 3: Estimates of relative egg-to-adult survival in the experiment and the litterature 7
Supplementary Table 4: Analysis of relative survival rate....................7
Supplementary Figure 1: Relative egg-to-adult survival rate per sex for each genotype....................8
Supplementary Figure 2: Relative egg-to-adult survival rates per generation for each genotype....................8
Development time at generation 5.................... 9
Supplementary Figure 3: Development time by genotype and sex. 9
Supplementary Table 5: Analysis of development time....................9
Deviation of genotypic proportion in the eggs in the experimental evolution.................... 10
Supplementary Table 6: Analysis of the deviation of genotypic proportion in the eggs relatively to random expectations....................10
Supplementary Figure 4: Relative deviation from random expectations in the eggs for each genotype and each generation....................10
Modelling the evolution of inversion frequencies.................... 11
Supplementary Table 7: Parameters of the individual-based model....................11
Supplementary Table 8: Goodness of fit to empirical data on 5 generations....................12
Supplementary Figure 5: Comparison of in silico experimental evolution to in vivo data 14
Supplementary Figure 6: Relative survival rate according to the duration of habitat availability....................15
Supplementary Figure 7: Evolution of the three genotypes proportions in simulations co-varying male reproductive success and environment. 16
Supplementary Figure 8: Frequency of the α rearrangement in simulations varying male reproductive success, the mean duration of habitat availability (Amean) and its variability (Avar). 17
Supplementary Figure 9: Evolution of the three genotypes proportions in simulations co-varying male reproductive success and environment in the medium density scenario. 18
Supplementary Figure 10: Outcome of simulations taking into account frequency-dependence effect on male reproductive success 19
Supplementary Figure 11: Time to fixation with sexually-varying fitness parameters....................20

Supplementary References.................... 21

Supplementary methods

Simulation model describe following the ODD procedure [1]

- Overview (Figure 3A, Supplementary Table 1, Supplementary Table 7)

Purpose: The purpose of the model was to evaluate how differences between genotypes in reproductive success and survival affect the evolution of the inversion genotype frequency in the experiment or in the wild and, more generally, the maintenance of polymorphism. *State variables and scales:* The model is based on individuals, which belong to the same population. Individuals are characterized by three state variables: stage (egg or adult), sex (male or female), inversion genotype (αα, αβ, ββ) and three traits: egg-to-adult survival, development time and reproductive success, which are dependent on sex and inversion genotype. Inversion alleles are inherited in a Mendelian fashion. *Process overview and scheduling:* The model consists of non-overlapping generations. Each time step is one generation. At each time step, two phases are processed in this order: reproduction of generation n-1, resulting in the population of eggs of the generation n, and egg-to-adult growth, resulting in the population of adults of generation n.

- Design concepts

Basic principles: The basic principle by which the model is constructed is a trade-off between two components of fitness, egg-to-adult survival and reproduction, which vary with the inversion genotype. Some scenarios include complementary features such as sex-specific parameters, trait-specific dominance including overdominance, frequency-dependence and environmental variation. *Adaptation:* Individuals have three adaptive traits, which are fully determined by their sex and genotype: egg-to-adult survival, development time and reproductive success. *Emergence:* Genotype frequencies at each generation, and thus inversion allelic frequencies, emerge as properties of the population from the relative fitness of the individuals. *Stochasticity:* All individual traits (survival, development time, relative reproductive success) and environmental variation (duration of habitat availability) are interpreted as probabilities, or are drawn from empirical probability distributions. Demographic stochasticity, somehow equivalent to a limited charge capacity, was included by randomly picking a subset of *K* eggs at the beginning of the growth phase. *Interaction:* Reproduction is modelled explicitly in relation to the number of available males and genotype-specific reproductive success. Duration of habitat availability interacts with individual development time and affects egg-to-adult survival. *Observation:* Genotype frequencies in the eggs and in the adults are the variable that we monitored across time steps.

- Details

Initialization: The model is initialized with N_0 adults at a sex-ratio of 50:50 and genotype proportions as observed in wild populations, i.e. in the experimental generation 0. N_0 is the product of *K*, the number of eggs kept for growth and S_0, the absolute survival rate.

Submodels - Reproduction: At each reproductive step, a pool of reproductive males was generated based on the distribution of adult males $\{N_{\alpha\alpha\text{-}m}\ ;\ N_{\alpha\beta\text{-}m}\ ;\ N_{\beta\beta\text{-}m}\}$, corrected by genotype-specific male reproductive success following $\{N_{\alpha\alpha\text{-}m} * T_{\alpha\alpha\text{-}m}; N_{\alpha\beta\text{-}m} * T_{\alpha\beta\text{-}m}; N_{\beta\beta\text{-}m} * T_{\beta\beta\text{-}m}\}$. This allowed modelling male reproductive success as a relative availability and propension for mating and took into account that males could reproduce many times in this species. For females, mixed paternity has been reported for *C. frigida* in only 5-10 % of the females [2], thus for simplicity, all females reproduced only once in the model. All females were considered successively in a random order. For each female, a male partner was randomly drawn from the pool of reproductive males, and the number of eggs laid by the pair was determined by the product between the number of eggs by a female and genotype-specific female fertility. Each egg inherited three state variables: (i) sex, which is determined by chance with no bias (sex-ratio= 0.5), (ii) genotype, which is assigned by

randomly drawing one allele from the mother, and one allele from the father, (iii) development time, which was calculated as the cubic root of three values randomly-drawn from a uniform distribution whose mean and range was determined by sex and genotype. This distribution was chosen as the one fitting best the measured experimental data of the development time. At the end of the reproductive step, a subset of randomly-picked *K* eggs proceeded to the growth step. This feature mimicked the experimental procedure and ensured a constant population size, being somehow equivalent to a carrying capacity.

Submodels – Growth: The outcome (survival or death) of each egg entering the growth phase was determined by chance with a survival probability determined by the product between global egg-to adult survival rate and relative genotype-sex specific survival. This step represents a form of "intrinsic mortality", observed even in favourable laboratory conditions. Yet, in the wild, *C. frigida*'s habitat is known to be temporary: the wrackbed can be removed monthly or bi-monthly by tides, and occasionally by storms [3]. To take this environmental effect into account, each surviving individual was attributed a habitat availability duration, randomly-drawn from a uniform distribution centred on A_{mean}, with width A_{var}. Variation in the duration of habitat availability between individuals can be interpreted as variation in the moment at which the egg was laid, as well as heterogeneity between wrackbeds. If development time exceeded the duration of habitat availability, the individual did not proceed to the adult stage. For models that do not consider such environmental effect, A_{mean} was taken as a very large value (30 days) and thus did not affect the total egg-to-adult survival.

- Simulated scenario and analysis

A mirror of the experimental evolution: To understand the dynamics of genotypic frequencies in the experimental system and to infer the parameters that could not be measured experimentally, such as male mating success, we ran the model over 5 generations, with K=1000 eggs, all parameters set to the measured experimental values and 30 simulations per set of parameters. We then explored several scenarios, (1) setting αα male mating success at 1 and varying male mating success of ββ between 0.05-1, αβ mating success being the mean of αα and ββ values (co-dominance), (2) setting αα and αβ mating success at 1 and varying male mating success of ββ between 0.05-1 (dominance), (3) setting αα male mating success at 1, and determining male mating success of αβ and ββ males as a function of αα-males frequency ($F_{\alpha\alpha\text{-m}}$) and two parameters (*FDC*, a coefficient of frequency dependence, and $t'_{\beta\beta\text{-}m}$, a fixed parameter) with the following equations:

$$(1)\ T_{\beta\beta-m} = t'_{\beta\beta-m} \cdot \left(1 - FDC \cdot (1 - \mathrm{F}_{\alpha\alpha-\mathrm{m}})\right)$$

$$(2)\ T_{\alpha\beta-m} = \frac{1 + t'_{\beta\beta-m}}{2} \cdot \frac{\left(1 - FDC \cdot (1 - \mathrm{F}_{\alpha\alpha-\mathrm{m}})\right)}{2}$$

For all scenarios, we compared the evolution of genotype proportions in the eggs and the adults over 5 generation to the empirically-observed evolution scenario. The fit of each simulation to empirical data was quantified by computing the normalized root-mean-squared error (nRMSE) for each genotypic proportion for generations 1 to 5. The average nRMSE over the 6 variables (αα/αβ/ββ proportions in the eggs and the adults), and over the 30 replicates, was taken as an index of fit, with the best predicting scenarios having the smallest values. Difference of mean nRMSE between the best scenarios was tested with a t-test based on the 30 replicates, corrected following [4]. Visualization of the evolution of frequencies was made with the R package *ggplot2* [5]

A model of natural populations: To expand the scope of the model to natural populations, the simulations were run over 200 generations with K= 10000 and 30 replicates per set of parameters. The null model was based on the parameters estimated in the experiment (and for male reproductive success, on parameters inferred from the best model fitted over 5 generations as described above). We then explored different sets of parameters that could represent a better approximation of natural conditions than experimental values (intermediate male reproductive success, limited substrate availability) or that present natural environmental variability (density, duration of habitat availability). This was done by following three scenarios that vary one parameter at a time and a scenario varying two parameters: (i) A scenario exploring survival variation with values estimated at medium and high density by Butlin *et al.* [6] (ii) A scenario varying male mating success of ββ between 0.05 and 1 in a co-dominance scenario, (iii) A scenario taking into account the length of habitat availability by varying A_{mean} between 7 and 15 days, (iv) a scenario setting male mating success of ββ at an intermediate value (0.5) and varying the duration of habitat availability from 7 to 15 days with two survival conditions, low (experimental) density and medium density. Genotypic proportions of the adults at the 200th generation were compared to values observed in wild populations [7,8] and at the 5th generation of our experiment. All simulations had reached stable genotypic proportions before the 100th generation. The proportion of the three genotypes under each scenario was visualized in ternary plots built with the R package *ggtern* [9]. Next, for the parameters that are more likely to vary in natural populations (male relative success, duration of habitat availability, density, variability in the duration of habitat availability), we explored under which combinations of realistic parameters polymorphism was maintained after 200 generations, what was the mean frequency of the inversion at equilibrium after 100 replicates, which portion of the parameter space lead to polymorphism and whether overdominance or sexual antagonism emerged for total fitness.

Generalization: To test more generally how polymorphism can be maintained by antagonistic pleiotropy in interaction with dominance/overdominance and sex-specific effects, we ran the same model based on a trade-off between survival and reproduction over 500 generations with K=10000 and 100 replicates. These simulations explored the whole theoretical parameter range for survival and reproduction (Supplementary Table 7), with either various scenarios of dominance, coded by the parameters *Hs*/*Ht*, or sex-specific effects with independent values for s_m/t_m and s_f/t_f ranging between 0 and 1. We surveyed the proportions of the simulations that led to maintenance of polymorphism vs. the simulations in which one of the genotypes got fixed, as well as the emerging mechanism at the level of total fitness (overdominance in one/both sex, sexual antagonism). Initial proportions were set to Hardy-Weinberg proportions, with the frequency of α being 0.5. The main features of the models remained the same, except that the effect of habitat availability/development time was not taken into account. In fact, the environmental effect on genotype-specific survival can be generalized in the survival parameter.

Supplementary Tables and Figures

Evolution of genotypic proportions in the experimental evolution

Supplementary Table 1: Number of samples genotyped and proportions of the three genotypes in the experiment.

Wild stands for wild-caught flies, *i. e.* the founders, generation 0. L stands for 90% Laminariaceae, 10% Fucaceae; F stands for 90% Fucaceae, 10% Laminariaceae.

				Adults				Eggs			
**population**	**replicate**	**generation**	**substrate**	**N**	**Proportions**			**N**	**Proportions**		
					**αα**	**αβ**	**ββ**		**αα**	**αβ**	**ββ**
	wild	0		94	0.09	0.54	0.37				
	CE01	1	L	47	0.21	0.60	0.19	51	0.18	0.61	0.22
	CE02	1	L	46	0.24	0.46	0.30	51	0.18	0.61	0.22
	CE03	1	L	42	0.24	0.64	0.12	51	0.18	0.61	0.22
	CE04	1	L	42	0.21	0.40	0.38	51	0.18	0.61	0.22
	CE07	1	F	48	0.08	0.67	0.25	51	0.18	0.61	0.22
	CE08	1	F	46	0.09	0.67	0.24	51	0.18	0.61	0.22
	CE09	1	F	40	0.18	0.50	0.33	51	0.18	0.61	0.22
	CE10	1	F	41	0.15	0.59	0.27	51	0.18	0.61	0.22
	CE01	2	L	48	0.31	0.46	0.23	43	0.33	0.44	0.23
	CE07	2	F	48	0.42	0.48	0.10	43	0.40	0.49	0.12
CE	CE01	3	L	41	0.34	0.61	0.05	45	0.44	0.49	0.07
	CE07	3	F	46	0.43	0.41	0.15	29	0.34	0.55	0.10
	CE01	4	L	46	0.43	0.54	0.02	45	0.51	0.42	0.07
	CE07	4	F	44	0.23	0.75	0.02	31	0.45	0.55	0.00
	CE01	5	L	48	0.46	0.52	0.02	28	0.57	0.39	0.04
	CE02	5	L	47	0.32	0.64	0.04				
	CE03	5	L	46	0.30	0.54	0.15				
	CE04	5	L	48	0.56	0.44	0.00				
	CE07	5	F	48	0.33	0.52	0.15	30	0.47	0.43	0.10
	CE08	5	F	47	0.49	0.47	0.04				
	CE09	5	F	47	0.43	0.49	0.09				
	CE10	5	F	48	0.40	0.54	0.06				
	wild	0		95	0.05	0.44	0.51				
	KA21	1	L	48	0.06	0.52	0.42	43	0.16	0.58	0.26
	KA23	1	L	47	0.06	0.62	0.32	43	0.16	0.58	0.26
	KA24	1	L	40	0.13	0.50	0.38	43	0.16	0.58	0.26
	KA25	1	L	43	0.07	0.63	0.30	43	0.16	0.58	0.26
	KA26	1	F	48	0.13	0.56	0.31	43	0.16	0.58	0.26
	KA27	1	F	48	0.04	0.48	0.48	43	0.16	0.58	0.26
	KA28	1	F	47	0.21	0.51	0.28	43	0.16	0.58	0.26
	KA30	1	F	42	0.07	0.48	0.45	43	0.16	0.58	0.26
	KA21	2	L	48	0.17	0.73	0.10	60	0.22	0.58	0.20
	KA27	2	F	46	0.20	0.65	0.15	36	0.22	0.53	0.25
KA	KA21	3	L	42	0.43	0.48	0.10	34	0.44	0.44	0.12
	KA27	3	F	45	0.33	0.64	0.02	30	0.40	0.47	0.13
	KA21	4	L	44	0.43	0.50	0.07	31	0.42	0.52	0.06
	KA27	4	F	42	0.33	0.64	0.02	35	0.43	0.49	0.09
	KA21	5	L	47	0.43	0.51	0.06	33	0.36	0.58	0.06
	KA23	5	L	44	0.57	0.36	0.07				
	KA24	5	L	41	0.34	0.61	0.05				
	KA25	5	L	48	0.25	0.73	0.02				
	KA26	5	F	43	0.47	0.47	0.07				
	KA27	5	F	47	0.45	0.49	0.06	45	0.40	0.47	0.13
	KA28	5	F	43	0.42	0.53	0.05				
	KA30	5	F	42	0.43	0.55	0.02				

Supplementary Table 2: Analysis of genotypic proportions

Generalized linear mixed model testing difference of genotypic proportions between generation 1 and 5 and either the effect of the population of origin or of the substrate.

Values are the z-values of the GLMM and p-value in brackets. The interaction between substrate and population was not tested given the size of the sample size (n= 4 replicates per generation, per substrate and per population)

	α	αα	αβ	ββ
generation	**10.6 (p<0.001)**	**8.8 (p<0.001)**	-1.4 (p=0.16)	**-7.9 (p<0.001)**
substrate	1.1 (p=0.27)	1.4 (p=0.16)	-0.49 (p=0.62)	-0.3 (p=0.78)
generation * substrate	-0.85 (p=0.40)	1.5 (p=0.14)	0.95 (p=0.34)	-0.55 (p=0.58)

	α	αα	αβ	ββ
generation	**8.2 (p<0.001)**	**6.9 (p<0.001)**	-1.3 (p=0.19)	**-6.5 (p<0.001)**
population	-3.6 (p<0.001)	-2.9 (p=0.004)	-0.9 (p=0.39)	3.3 (p=0.001)
generation * population	2.8 (p=0.005)	2.6 (p=0.01)	0.8 (p=0.41)	-2.3 (p=0.02)

Egg-to adult relative survival in the experimental evolution

Supplementary Table 3: Estimates of relative egg-to-adult survival in the experiment and the litterature

		$S_{\alpha\alpha\text{-}f}$	$S_{\alpha\beta\text{-}f}$	$S_{\beta\beta\text{-}f}$	$S_{\alpha\alpha\text{-}m}$	$S_{\alpha\beta\text{-}m}$	$S_{\beta\beta\text{-}m}$
Experimental data (32 replicates)	mean [sd]	0.80 [0.36]	1.01 [0.22]	1.12 [0.7]	0.88 [0.47]	1.08 [0.29]	0.96 [0.58]
	(normalized for comparison)	0.79	1	1.11	0.81	1	0.88
From Butlin *et al* [6]	Low density	0.87	1	1.09	0.83	1	0.73
	Medium density	0.52	1	0.63	0.40	1	0.35
	High density	0.56	1	0.34	0.33	1	0.32
From Gilburn *et al* [10]	mean [sd]	0.64 [0.11]	1.18 [0.11]	0.91 [0.12]	0.64 [0.11]	1.18 [0.11]	0.91 [0.12]
	(No sex-specific data)	0.54	1	0.77	0.54	1	0.77

Supplementary Table 4: Analysis of relative survival rate

Linear mixed models testing the effect of genotype, sex, substrate or population on the

Note: The effect of generation is driven by the variation in ββ survival (Supplementary Figure 2), which is somehow lower in the last generations. Yet, the effect of generation is difficult to test and may arise from stochasticity because sample size is small (4 replicates per generations) and because survival rates at generation 3 to 5 are not strictly reliable given the low absolute numbers of BB found in the eggs or the adults after generation 3.

	Df	F	P
**genotype**	2	4.7	**0.01**
**sex**	1	0	0.99
**substrate**	1	0.06	0.8
**population**	1	0.87	0.35
**generation**	1	0.7	0.4
**genotype * sex**	2	1.5	0.21
**genotype * substrate**	2	0.78	0.46
**genotype * population**	2	0.43	0.65
**genotype * generation**	2	11.2	**<0.001**

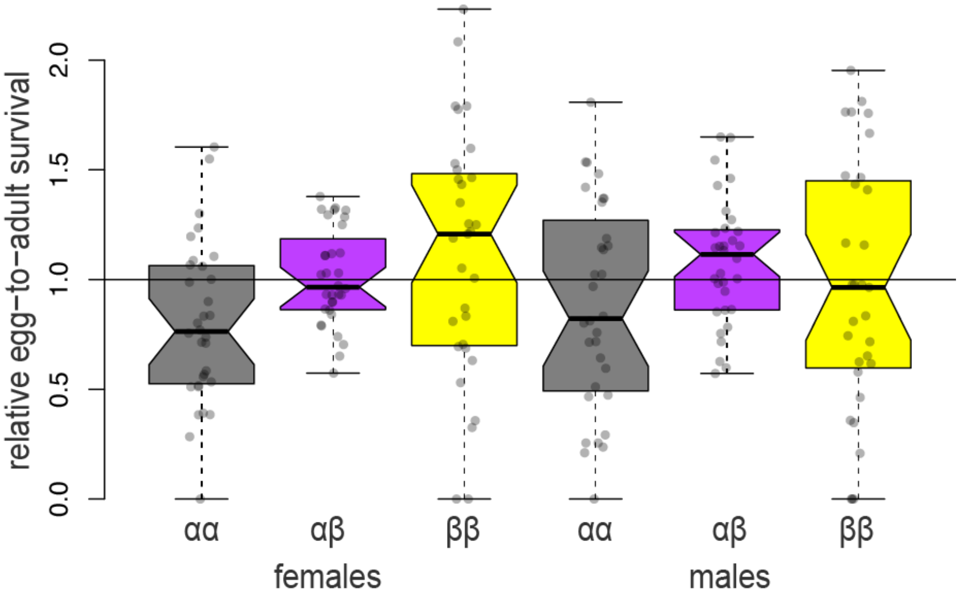

Supplementary Figure 1: Relative egg-to-adult survival rate per sex for each genotype
Neither sex nor the interaction sex:genotype significantly affect the relative survival rate. Pairwise t-test do not show significant differences between mean survival rates because of the large variance heterogeneity. Central line represents the median, boxes are the quartiles with notches representing the confident interval of the median, and whiskers expand to maximum values. Overlapping points are individual values.

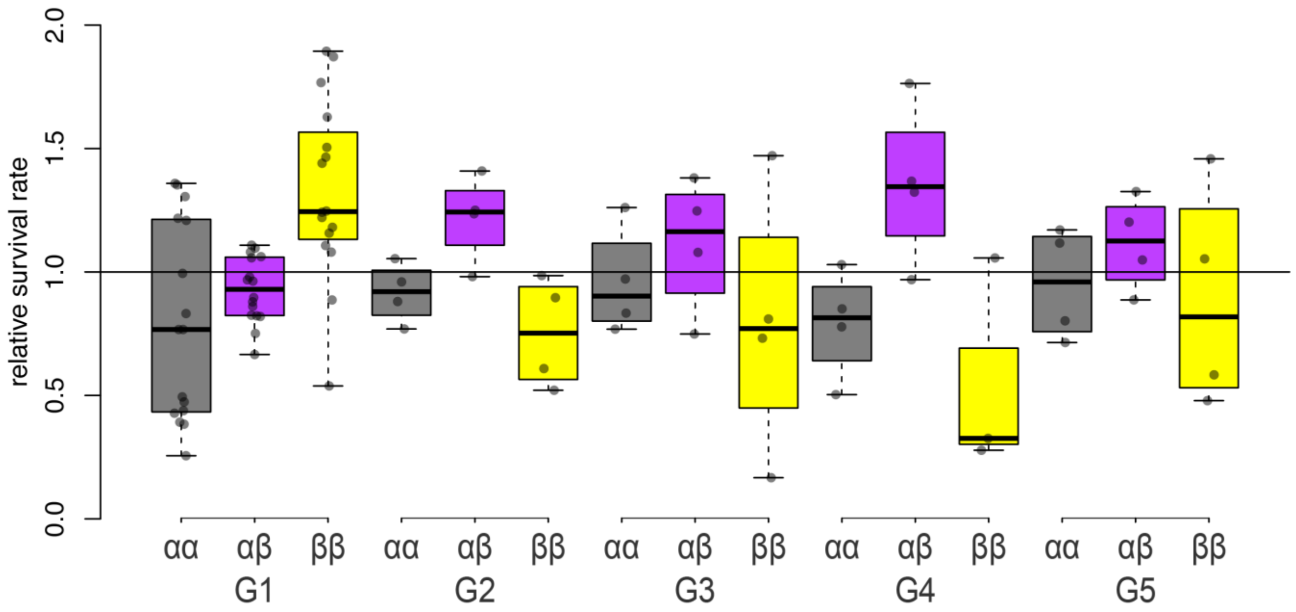

Supplementary Figure 2: Relative egg-to-adult survival rates per generation for each genotype
Central line represents the median, boxes are the quartiles and whiskers expand to maximum values. Overlapping points are individual values. Note: Although there seems to be variation in survival rate between generations and the test suggests a significant genotype*generation effect on survival rate (Supplementary Table 2), there is no clear trend and much stochasticity may come from the fact that generations 2 to 5 include four replicates and survival rates at generation 3 to 5 are not extremely reliable given the low absolute numbers of BB found in the eggs or the adults after generation 4. G stands for generation.

Development time at generation 5

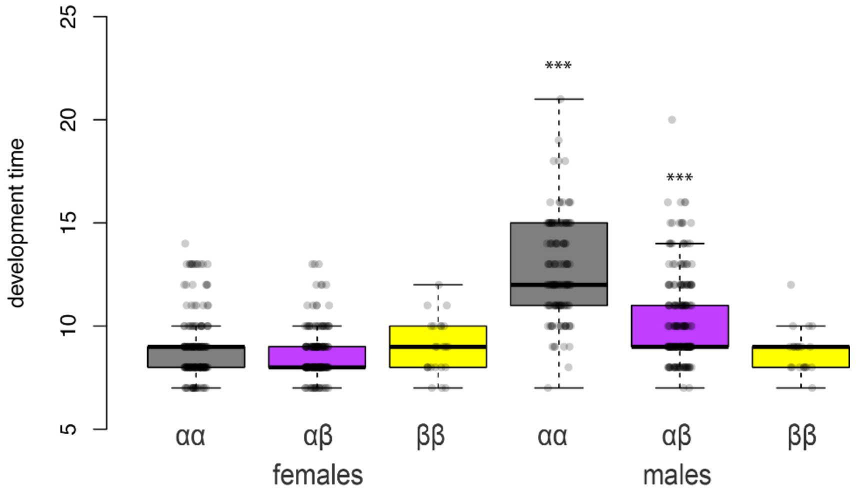

Supplementary Figure 3: Development time by genotype and sex.
Time is counted as the number of days between egg laying and adult emergence. Data are drawn from the 5th generation, with the 16 replicates pooled. *Coelopa frigida* were raised on either Fucaceae or Laminariaceae, at low densities and constant temperature 25°C. Development time is expected to be longer under a higher density or at lower temperature. For instance in a similar experiment at 15°C, the females and the first males generally emerged after around 15 days while the last males took more than 30 days. Central line represents the median, boxes are the quartiles and whiskers expand to 1.5 times the interquartile. Stars represent significant differences ($p<0.001$) from all other groups in a glmm pairwise test.

Supplementary Table 5: Analysis of development time
Generalized linear mixed model testing the effect of sex, genotype and substrate on development time.
Box identity (replicate of the experimental evolution) was taken as random factor and we applied a Poisson transformation. X^2 and p-values come from comparisons between nested models with X^2-tests.

	**variable**	**DF**	**X^2**	**P-value**	
All samples	sex	3	100.3	<0.001	
	genotype	4	26.2	<0.001	
	substrat	3	0.05	0.81	
	sex* genotype	7	157.37	<0.001	(best model)
Females	genotype	4	1.1	0.57	
	substrat	3	0.04	0.85	
	genotype * substrat	7	0.72	0.87	
Males	genotype	4	55.7	<0.001	(best model)
	substrat	3	0.19	0.66	
	genotype * substrat	7	1.25	0.74	

Deviation of genotypic proportion in the eggs in the experimental evolution

Supplementary Table 6: Analysis of the deviation of genotypic proportion in the eggs relatively to random expectations

Linear mixed models testing the effect of genotype, sex, substrate or population on genotypic frequencies in the eggs relatively to Hardy-Weinberg proportions of the previous generation

Random factor is the identity of the replicate box. Note: The effect of the interaction between genotype and generation is driven by the lower excess of αα at generation 3-4-5 compared to generation 1-2 (Supplementary Figure 4). However, such effect of generation should be interpreted with much caution since sample size is 4 replicates par generations and deviations at generation 3 to 5 are not as reliable as at earlier generation given the low absolute numbers of BB found in the eggs or the adults.

	Df	F	P
genotype	2	24.9	<0.001
generation	1	10.5	0.002
population	1	1.93	0.17
substrate	1	0.03	0.87
genotype * generation	2	8.34	<0.001
genotype * population	2	0.07	0.92
genotype * substrate	2	0.08	0.92

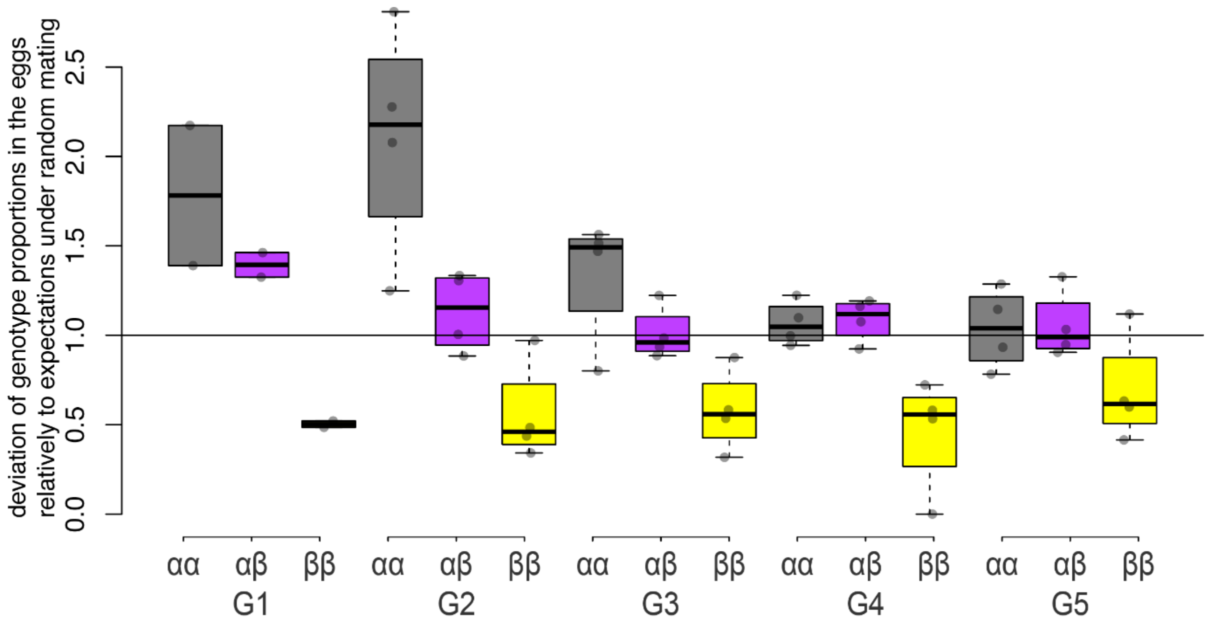

Supplementary Figure 4: Relative deviation from random expectations in the eggs for each genotype and each generation

Central line represents the median, boxes are the quartiles and whiskers expand to maximum values. Overlapping points are individual values. G stands for generation.

Modelling the evolution of inversion frequencies

Supplementary Table 7: Parameters of the individual-based model

	Parameter name	Description	Empirical values *[explored values]* used for simulations of the experiment	Default values [explored values] used for simulations in wild populations	theoretical models
All_models	*G*	Number of generations	5	200	500
	K	Number of eggs kept per generation	1,000	10,000	10,000
	E	Number of eggs per female	70	70	70
	V	Global egg-to-adult Viability	0.3	0.3	0.3
	R	Sex-ratio	0.5	0.5	0.5
	N0	Number of adults at generation 0	300	3,000	3,000
	P0-αα-ad	Proportions of αα adults at generation 0	0.07	0.07	0.25
	P0-αβ-ad	Proportions of αβ adults at generation 1	0.49	0.49	0.5
	P0-ββ-ad	Proportions of ββ adults at generation 2	0.44	0.44	0.25
	Sααf	Relative egg-to-adult survival of αα females	0.71	0.71 *[0.71; 0.52; 0.56]*	1-S_f
	Sαβf	Relative egg-to-adult survival of αβ females	0.9	0.90 *[0.90; 1.0; 1.0]*	1-S_f*H_s
	Sββf	Relative egg-to-adult survival of ββ females	1.0	1 *[1.0; 0.63; 0.34]*	1
	Sααm	Relative egg-to-adult survival of αα males	0.81	0.81 *[0.81; 0.40; 0.33]*	1-S_m
	Sαβm	Relative egg-to-adult survival of αβ males	1.0	1.0 *[1.0; 1.0; 1.0]*	1-S_m*H_s
	Sββm	Relative egg-to-adult survival of ββ males	0.88	0.88 *[0.88; 0.35; 0.32]*	1
	Tααf	Relative fecundity of αα females	1.0	1.0	1
	Tαβf	Relative fecundity of αβ females	0.97	0.97	1-t_f*H_t
	Tββf	Relative fecundity of ββ females	0.87	0.87	1-t_f
	Tααm	Relative reproductive success of αα males	1.0 *[1.0]*	1.0 *[1.0]*	1
	Tαβm	Relative reproductive success of αβ males	*[0.525 : 1.0]*	0.5 *[0.55 : 1.0]*	1-t_m*H_t
	Tββm	Relative reproductive success of ββ males	*[0.05 : 1.0]*	0.1 *[0.1 : 1.0]*	1-t_m
with_freq-dpdce effect	*FDC*	Frequency-dependance coefficient	*[0 : 0.9]*	0.9	0
	tααm	Relative reproductive success of αα males	1.0 *[1.0]*	1.0	
	tαβm	Relative reproductive success of αβ males	*[0.55 : 1.0]*	0.8	
	tββm	Relative reproductive success of ββ males	*[0.1 : 1.0]*	0.6	
with environmental effect	*Df*	Development time of females (in days)	8.8	8.8	8.8
	Dααm	Development time of αα males (in days)	12.8	12.8	12.8
	Dαβm	Development time of αβ males (in days)	10.3	10.3	10.3
	Dββm	Development time of ββ males (in days)	8.7	8.7	8.7
	DCV	Variation coefficient of development time	0.5	0.5	0.5
	Amean	Duration of habitat availability (in days)	30	30 *[7 : 20]*	30
	ACV	Variation coefficient of the duration of habitat availability	1	2 *[0 : 10]*	1
for theoretical model	s / s_f / s_m	survival difference between homozygotes (for both sexes, females or males)			[0 : 1]
	t / t_f / t_m	reproductive difference between homozygotes (for both sexes, females or males)			[0 : 1]
	H_s	dominance in heterozygotes for survival			[-0,25, 0, 0.25, 0.5]
	H_t	dominance in heterozygotes for reproduction			[-0,25, 0, 0.25, 0.5]

Supplementary Table 8: Goodness of fit to empirical data on 5 generations.

Fit is measured by the nrmse (normalized mean squared error) on the genotypic proportions in the adults and eggs. The difference of mean nrmse between all scenarios by a pairwise t-test between all the scenario (30 replicates per scenario), corrected for multiple testing following Benjamini & Hochberg. Below we present only the comparison to the best 6 models, selected as best based on their goodness of fit and different parametrizations explored. *** stands for p<0.01

		Scenario	$T_{\alpha\alpha\text{-}m}$	$T_{\alpha\beta\text{-}m}$	$T_{\beta\beta\text{-}m}$	FDC	mean nrmse	p-value of t-test on the mean nrmse difference between each scenario the selected best 6 scenarios					
								Exp BB0.1	Exp BB0.05	Exp Domin BB0.05	Exp BB0.4 freq0.9	Exp BB0.4 freq0.8	Exp BB0.6 freq0.9
BEST SCENARIOS FITTING EMPIRICAL OBSERVATIONS													
**Without frequency-dependance**	**Co-dominance**	exp_BB0.1	1	0.55	0.1	0	***28***		0.53	0.34	0.50	0.41	0.78
		exp_BB0.05	1	0.525	0.05	0	***28***	0.53		0.10	0.97	0.86	0.35
	**dominance**	exp_domin_BB0.05	1	1	0.05	0	***33***	0.34	0.10		0.10	0.07	0.51
**With strong frequency-dependance effect**		exp_BB0.4_freq0.9	1	0.7	0.4	0.9	***26***	0.50	0.97	0.10		0.89	0.34
		exp_BB0.4_freq0.8	1	0.7	0.4	0.8	***26***	0.41	0.86	0.07	0.89		0.27
		exp_BB0.6_freq0.9	1	0.8	0.6	0.9	***30***	0.78	0.35	0.51	0.34	0.27	
OTHER SCENARIOS WITH REDUNDANT PARAMETRIZATION AND EQUIVALENT FIT													
**Equivalent to the case BB=0.1 and no frequency-dependence (very low BB success)**		exp_BB0.1_freq0.2	1	0.55	0.1	0.2	***25***	0.21	0.55	**0.02**	0.58	0.68	0.12
		exp_BB0.1_freq0.3	1	0.55	0.1	0.3	***25***	0.29	0.68	**0.04**	0.71	0.82	0.17
		exp_BB0.1_freq0.4	1	0.55	0.1	0.4	***28***	0.52	1.00	0.10	0.98	0.87	0.35
		exp_BB0.1_freq0.5	1	0.55	0.1	0.5	***26***	0.35	0.77	0.05	0.80	0.91	0.22
		exp_BB0.1_freq0.6	1	0.55	0.1	0.6	***27***	0.64	0.87	0.15	0.85	0.74	0.45
		exp_BB0.1_freq0.1	1	0.55	0.1	0.1	***28***	0.93	0.59	0.29	0.57	0.47	0.71
		exp_BB0.1_freq0.7	1	0.55	0.1	0.7	***33***	0.25	0.07	0.85	0.06	**0.04**	0.39
		exp_BB0.2_freq0.6	1	0.6	0.2	0.6	***27***	0.46	0.92	0.08	0.95	0.94	0.30
		exp_BB0.2_freq0.5	1	0.6	0.2	0.5	***28***	0.97	0.55	0.32	0.53	0.43	0.76
		exp_BB0.2_freq0.8	1	0.6	0.2	0.8	***28***	0.71	0.81	0.18	0.78	0.67	0.51
		exp_BB0.2_freq0.7	1	0.6	0.2	0.7	***29***	0.85	0.40	0.45	0.38	0.31	0.93
		exp_BB0.2_freq0.4	1	0.6	0.2	0.4	***30***	0.64	0.26	0.64	0.25	0.19	0.85
		exp_BB0.2_freq0.9	1	0.6	0.2	0.9	***31***	0.71	0.31	0.57	0.29	0.23	0.93
		exp_BB0.2_freq0.3	1	0.6	0.2	0.3	***31***	0.61	0.24	0.67	0.23	0.18	0.82
		exp_BB0.2_freq0.2	1	0.6	0.2	0.2	***33***	0.20	**0.05**	0.75	**0.05**	**0.03**	0.32
**Equivalent to the case with strong-frequency dependence effect (and higher BB success)**		exp_BB0.6_freq0.8	1	0.8	0.6	0.8	***34***	0.10	**0.02**	0.50	**0.02**	**0.01**	0.17
		exp_BB0.4_freq0.7	1	0.7	0.4	0.7	***34***	0.17	**0.04**	0.69	**0.04**	**0.03**	0.28
		exp_BB0.8_freq0.9	1	0.9	0.8	0.9	***34***	0.20	0.05	0.76	**0.05**	**0.03**	0.33
OTHER SCENARIOS EXPLORED													
**Combination of parameters with significantly-reduced fit to empirical observations (by comparison with best models)**		exp_BB0.1_freq0.8	1	0.55	0.1	0.8	***35***	0.09	**0.02**	0.49	**0.02**	**0.01**	0.17
		exp_BB0.2_freq0.1	1	0.6	0.2	0.1	***35***	0.07	**0.01**	0.43	**0.01**	**0.01**	0.14
		exp_BB0.4_freq0.6	1	0.7	0.4	0.6	***37***	**0.01**	***	0.10	***	***	**0.02**
		exp_domin_BB0.1	1	1	0.1	0	***37***	**0.01**	***	0.15	***	***	**0.03**
		exp_BB0.1_freq0.9	1	0.55	0.1	0.9	***38***	**0.02**	***	0.15	***	***	**0.03**
		exp_BB0.2	1	0.6	0.2	0	***38***	**0.01**	***	0.11	***	***	**0.02**
		exp_BB0.2_freq0.0	1	0.6	0.2	0	***39***	***	***	**0.04**	***	***	**0.01**
		exp_BB1.0_freq0.9	1	1	1	0.9	***40***	***	***	**0.02**	***	***	***
		exp_BB0.6_freq0.7	1	0.8	0.6	0.7	***40***	***	***	**0.01**	***	***	***

exp_BB0.4_freq0.5	1	0.7	0.4	0.5	***41***	***	***	0.01	***	***	***
exp_BB0.8_freq0.8	1	0.9	0.8	0.8	***44***	***	***	***	***	***	***
exp_domin_BB0.2	1	1	0.2	0	***44***	***	***	***	***	***	***
exp_BB0.4_freq0.4	1	0.7	0.4	0.4	***45***	***	***	***	***	***	***
exp_BB0.4_freq0.3	1	0.7	0.4	0.3	***48***	***	***	***	***	***	***
exp_BB1.0_freq0.8	1	1	1	0.8	***49***	***	***	***	***	***	***
exp_BB0.8_freq0.7	1	0.9	0.8	0.7	***50***	***	***	***	***	***	***
exp_BB0.3	1	0.65	0.3	0	***51***	***	***	***	***	***	***
exp_BB0.6_freq0.6	1	0.8	0.6	0.6	***51***	***	***	***	***	***	***
exp_BB0.6_freq0.5	1	0.8	0.6	0.5	***52***	***	***	***	***	***	***
exp_domin_BB0.3	1	1	0.3	0	***53***	***	***	***	***	***	***
exp_BB0.4_freq0.2	1	0.7	0.4	0.2	***53***	***	***	***	***	***	***
exp_BB0.4_freq0.1	1	0.7	0.4	0.1	***55***	***	***	***	***	***	***
exp_BB0.8_freq0.6	1	0.9	0.8	0.6	***58***	***	***	***	***	***	***
exp_domin_BB0.4	1	1	0.4	0	***60***	***	***	***	***	***	***
exp_BB1.0_freq0.7	1	1	1	0.7	***60***	***	***	***	***	***	***
exp_BB0.6_freq0.4	1	0.8	0.6	0.4	***60***	***	***	***	***	***	***
exp_BB0.4_freq0.0	1	0.7	0.4	0	***62***	***	***	***	***	***	***
exp_BB0.4	1	0.7	0.4	0	***63***	***	***	***	***	***	***
exp_BB0.8_freq0.5	1	0.9	0.8	0.5	***65***	***	***	***	***	***	***
exp_BB0.6_freq0.3	1	0.8	0.6	0.3	***67***	***	***	***	***	***	***
exp_domin_BB0.5	1	1	0.5	0	***69***	***	***	***	***	***	***
exp_domin_BB0.6	1	1	0.6	0	***70***	***	***	***	***	***	***
exp_BB0.6_freq0.2	1	0.8	0.6	0.2	***70***	***	***	***	***	***	***
exp_BB1.0_freq0.6	1	1	1	0.6	***70***	***	***	***	***	***	***
exp_BB0.5	1	0.75	0.5	0	***73***	***	***	***	***	***	***
exp_BB0.8_freq0.4	1	0.9	0.8	0.4	***74***	***	***	***	***	***	***
exp_BB1.0_freq0.5	1	1	1	0.5	***79***	***	***	***	***	***	***
exp_BB0.6_freq0.1	1	0.8	0.6	0.1	***83***	***	***	***	***	***	***
exp_BB0.8_freq0.3	1	0.9	0.8	0.3	***84***	***	***	***	***	***	***
exp_BB0.6	1	0.8	0.6	0	***85***	***	***	***	***	***	***
exp_domin_BB0.7	1	1	0.7	0	***86***	***	***	***	***	***	***
exp_BB0.6_freq0.0	1	0.8	0.6	0	***86***	***	***	***	***	***	***
exp_BB0.8_freq0.2	1	0.9	0.8	0.2	***86***	***	***	***	***	***	***
exp_BB1.0_freq0.4	1	1	1	0.4	***87***	***	***	***	***	***	***
exp_BB1.0_freq0.3	1	1	1	0.3	***89***	***	***	***	***	***	***
exp_BB0.8_freq0.1	1	0.9	0.8	0.1	***94***	***	***	***	***	***	***
exp_domin_BB0.8	1	1	0.8	0	***95***	***	***	***	***	***	***
exp_BB0.7	1	0.85	0.7	0	***96***	***	***	***	***	***	***
exp_BB0.8	1	0.9	0.8	0	***99***	***	***	***	***	***	***
exp_BB0.9	1	0.95	0.9	0	***99***	***	***	***	***	***	***
exp_BB1.0_freq0.2	1	1	1	0.2	***101***	***	***	***	***	***	***
exp_BB0.8_freq0.0	1	0.9	0.8	0	***102***	***	***	***	***	***	***
exp_domin_BB0.9	1	1	0.9	0	***105***	***	***	***	***	***	***
exp_BB1.0_freq0.1	1	1	1	0.1	***105***	***	***	***	***	***	***
exp_BB1.0	1	1	1	0	***105***	***	***	***	***	***	***
exp_domin_BB1.0	1	1	1	0	***111***	***	***	***	***	***	***
exp_BB1.0_freq0.0	1	1	1	0	***117***	***	***	***	***	***	***

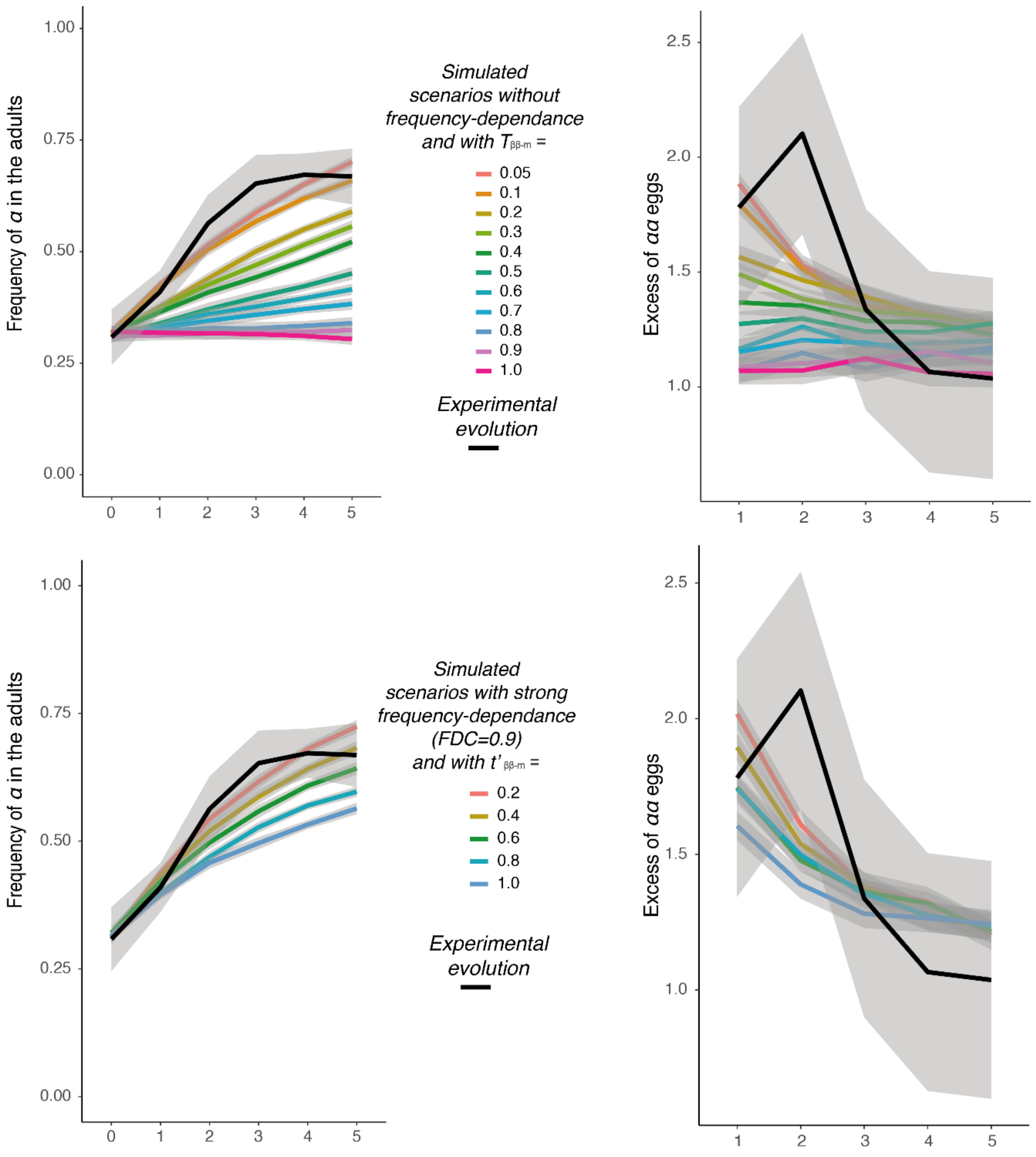

Supplementary Figure 5: Comparison of in silico experimental evolution to in vivo data

Evolution of the frequency of the α rearrangement and the deviation of αα in the eggs across 5 generations. The two-best scenario (as scored by nRMSE) are TBB-m = 0.1 or 0.05 without frequency dependence, and FDC=0.9 and TBB-m = 0.4 or 0.6, with frequency-dependance. Data are smoothed using a loess method across 4 replicates per generation for experimental data and across 30 replicates per generation and per set of parameters for simulated data, and standard error bounds are represented by the grey shade.

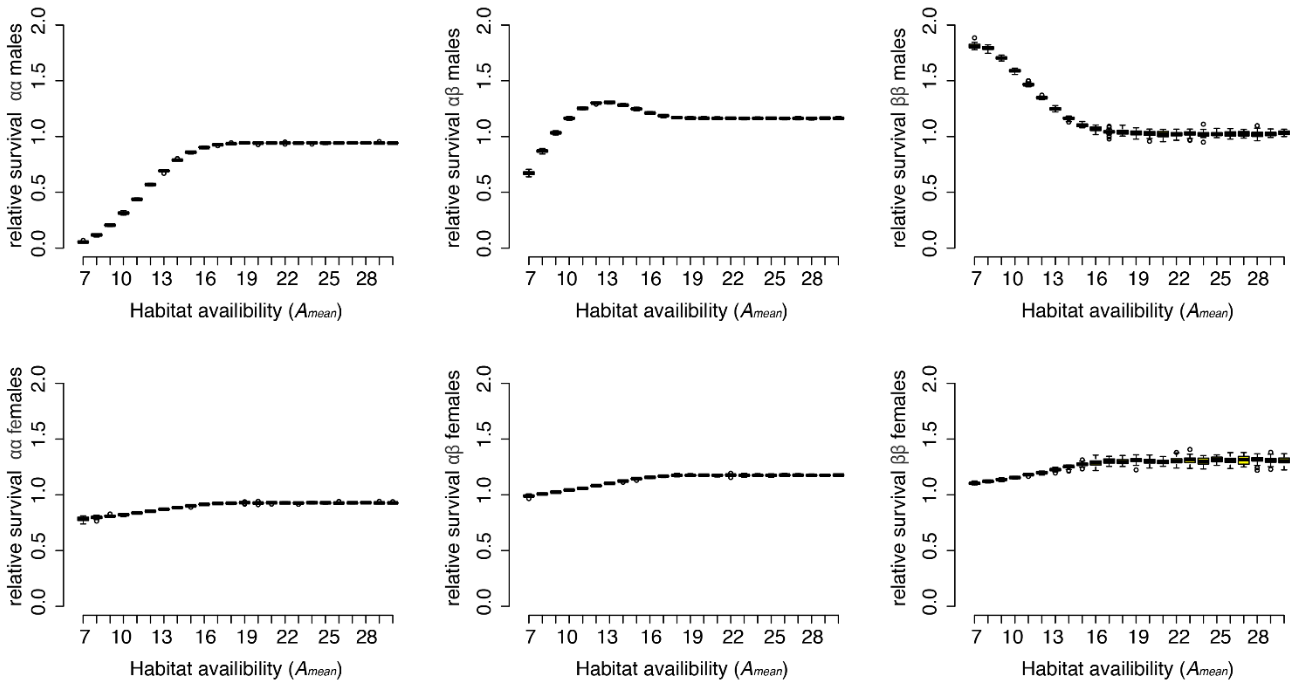

Supplementary Figure 6: Relative survival rate according to the duration of habitat availability. This value is an estimate of the relative survival rate includes both the intrinsic egg-to-adult survival rate and the effect of the environment. It is calculated as the deviation between the proportions in the adults and in the eggs. Central line represents the median, boxes are the quartiles and whiskers expand to 1.5 times the interquartile and points are the outliers.

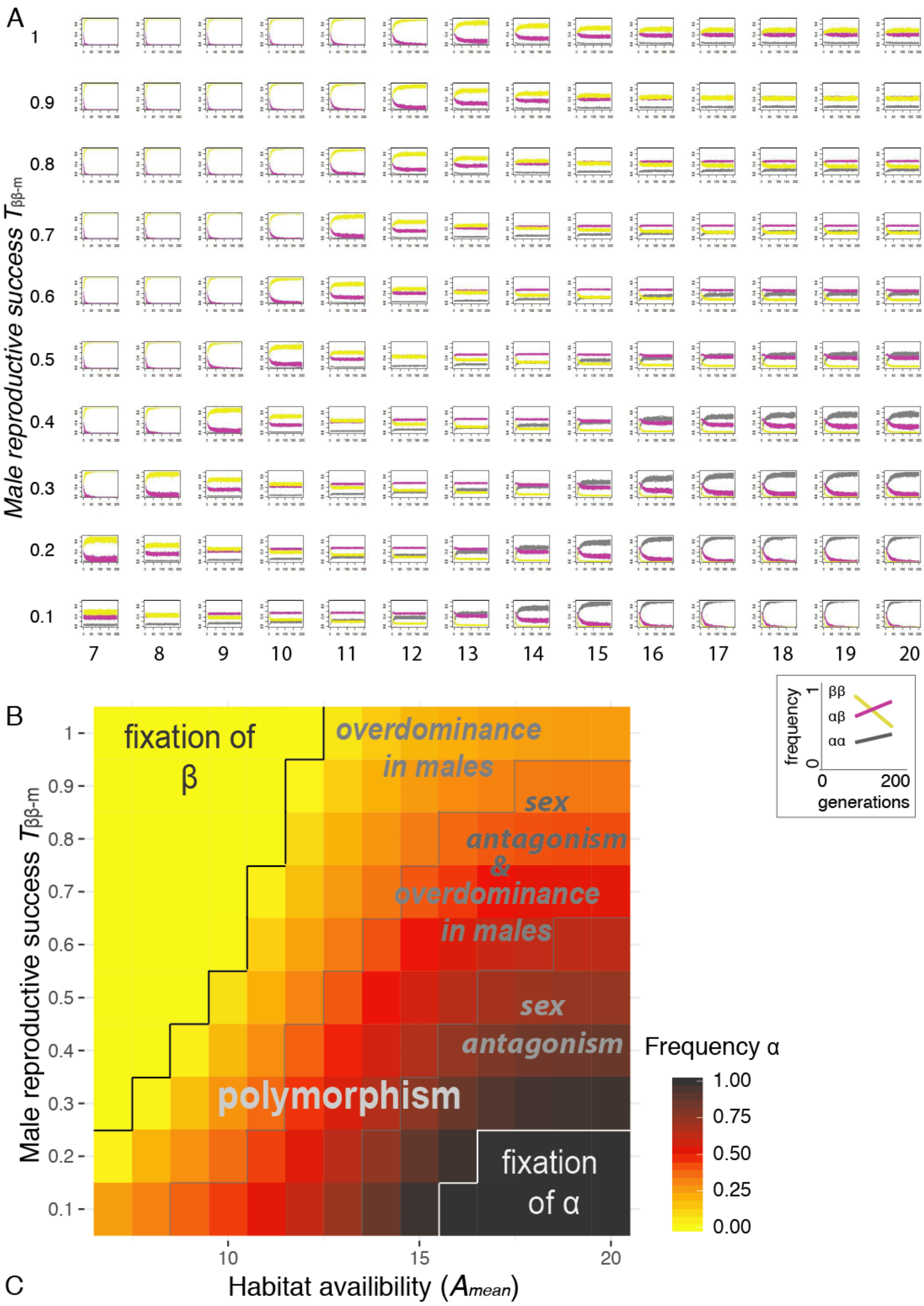

Supplementary Figure 7: Evolution of the three genotypes proportions in simulations co-varying male reproductive success and environment.

Each subplot on panel (A) represents the evolution of the frequency of the three genotypes (ββ: yellow, αβ: purple, αα: grey) as a function of time (generations 0 to 200) as outline in the insert in the lowest left corner. The disposition of the plots is a mirror of Figure 5A (recalled in panel B), that represent the frequency of α allele at equilibrium (after 200 generations). Each row represents a different value of Tββ-m (male reproductive success of ββ), from 1.0 (= Tαα-m) to 0.1 (= tenfold lower than Tαα-m). Each column represents a different value of the duration of habitat availability (A mean). Variability in the duration of the environment is fixed at Avar =2. Relative survival rate (S parameter) corresponds to values as estimated in the experiment (low density scenario)

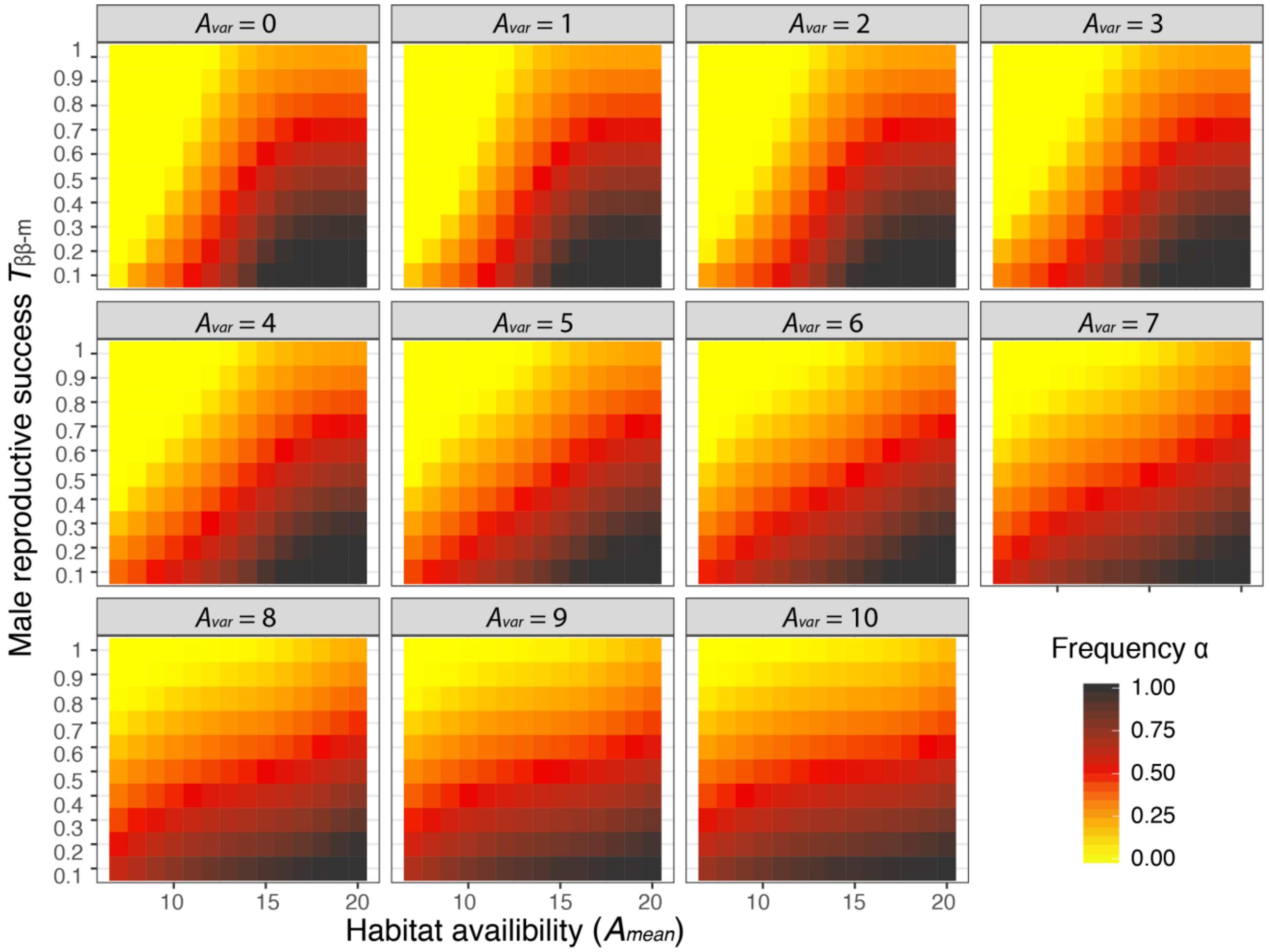

Supplementary Figure 8: Frequency of the α rearrangement in simulations varying male reproductive success, the mean duration of habitat availability (Amean) and its variability (Avar).

Each subplot represents the mean frequency of alpha after 200 generations across 100 replicates in the parameter space defined by the y-axis, male reproductive success (Tββ-m), and the x-axis, mean duration of habitat availability (Amean). Yellow areas correspond to a fixation of the β allele, black area to the fixation of α allele, and orang/red/brown area to the persistence of polymorphism, as shown on Figure 5A. With increasing variability in the duration of habitat availability (Avar, expressed in days), we observed a wider set of parameters for which polymorphism is maintained and α frequency remains at intermediate values.

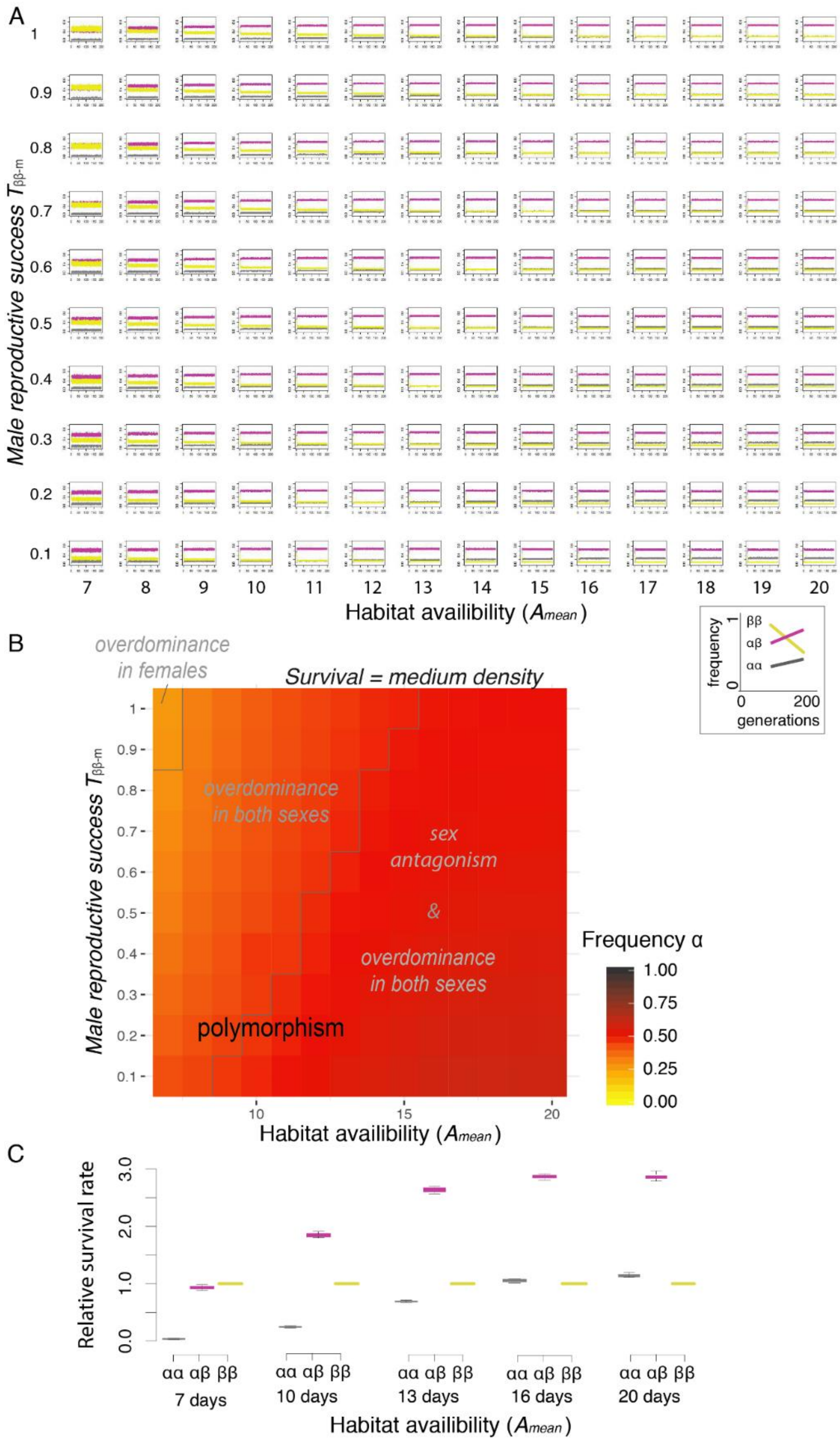

Supplementary Figure 9: Evolution of the three genotypes proportions in simulations co-varying male reproductive success and environment in the medium density scenario.

Each subplot on panel (A) represents the evolution of the frequency of the three genotypes (ββ: yellow, αβ: purple, αα: grey) as a function of time (generations 0 to 200) as outline in the insert in the lowest left corner. The disposition of the plots is a mirror of panel B that represent the frequency of α allele at equilibrium (after 200 generations). Variability in the duration of the environment is fixed at Avar =2. Relative survival rate (S parameter) corresponds to values estimates at medium density by Butlin et al [6]. This relative survival rate is further affected by the possibility to reach adulthood, as modelled with the

interaction between development time and the availability of the habitat. Panel C thus represent the Overall relative survival rate in males, after the effect of environment, for the three genotypes in those simulations varying the duration of habitat availability (normalized relatively to Sββ-m = 1). Bars represent the median and whiskers are 1.5 the interquartile. Boxes are not visible.

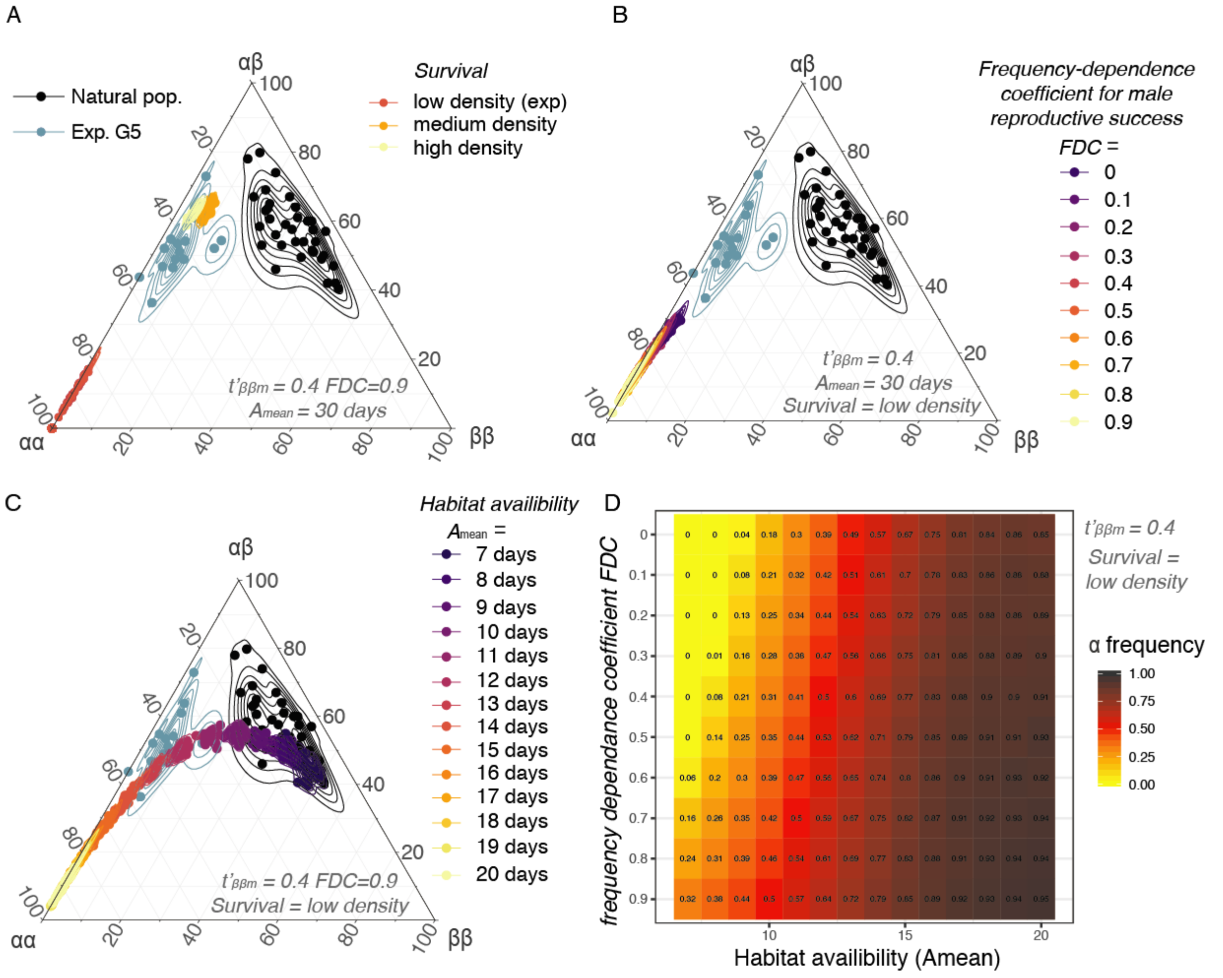

Supplementary Figure 10: Outcome of simulations taking into account frequency-dependence effect on male reproductive success

(A-C)Ternary plots comparing the proportions of the three genotypes in natural populations [7,8], after the 5th generation of our laboratory experiment and at the equilibrium after 200 generations of simulations taking into account a effect of the frequency of αα males on male reproductive success. In a few words, the relative reproductive success of αα (large) males is reduced when they are more frequent. Since this parameter is fixed to 1, this translate into the reproductive success of ββ males increasing with the frequency of αα males. The set of parameters is inspired by the best model fitting experimental data (see Supplementary Table 8 & Figure 5), i. e. FDC=0.9 and t'ββ-m =0.4. We explore scenarios varying (A) the effect of density, and the related relative survival rate, (B) the range of values for the frequency-dependence parameter on male relative reproductive success (FDc, t'ββ-m is fixed to 0.4) C) the effect of a limited duration of the habitat availability (Amean = [7-20 days], Avar = 2 days). (D) Polymorphism persistence and frequency of the inversion at equilibrium in simulations co-varying the duration of habitat availability (which modulate male relative survival) and the strength of the frequency-dependence impact on male reproductive success.

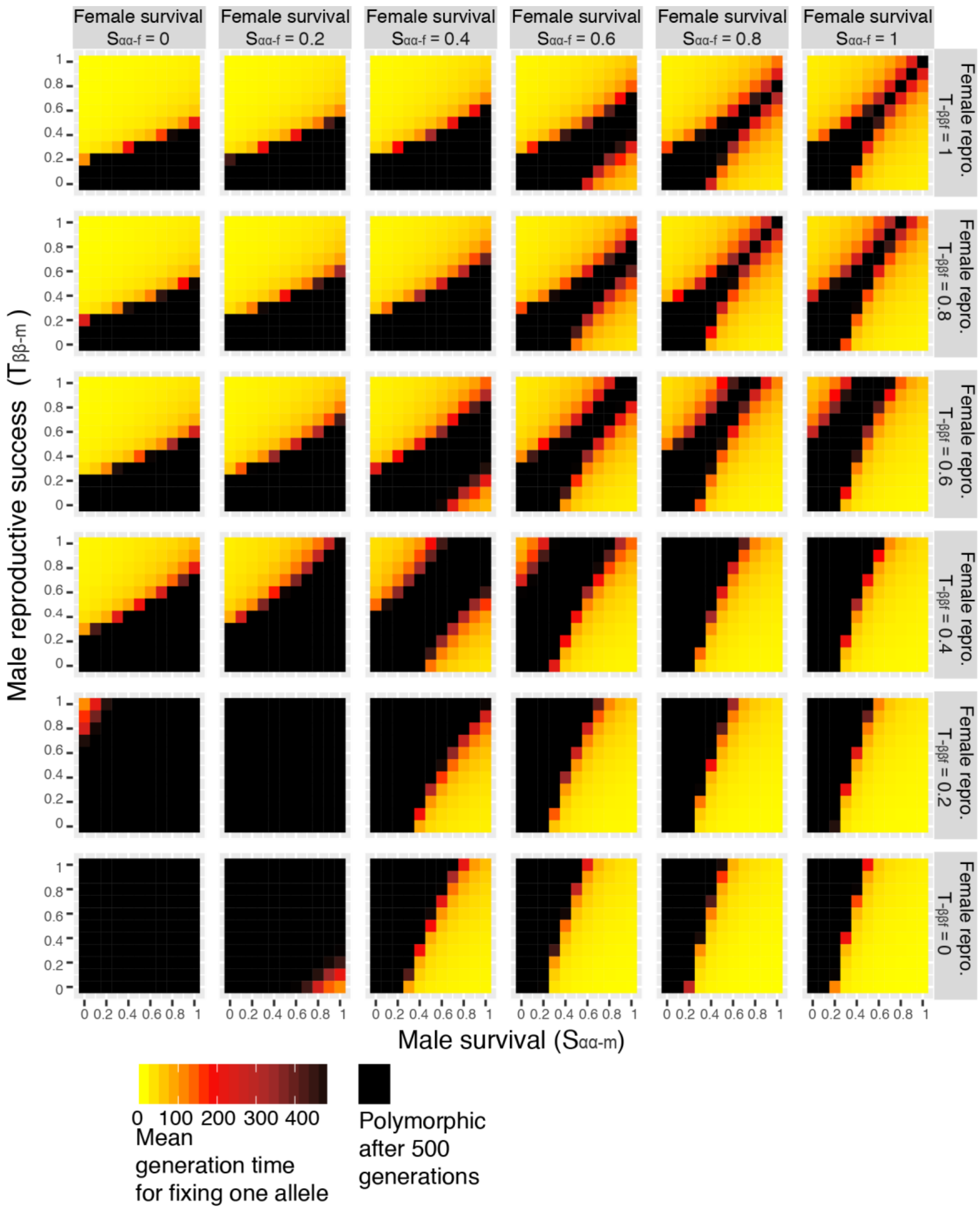

Supplementary Figure 11: Time to fixation with sexually-varying fitness parameters

Supplementary References

1. Grimm, V. *et al.* The ODD protocol: a review and first update. *Ecological modelling* **221**, 2760–2768 (2010).
2. Day, T., Miles, S., Pilkington, M. & Butlin, R. Differential mating success in populations of seaweed flies (Coelopa frigida). *Heredity* **58**, 203–212 (1987).
3. Dobson, T. Studies on the biology of the kelp-fly Coelopa in Great Britain. *Journal of Natural History* **8**, 155–177 (1974).
4. Benjamini, Y. & Hochberg, Y. Controlling the false discovery rate: a practical and powerful approach to multiple testing. *Journal of the royal statistical society. Series B (Methodological)* 289–300 (1995).
5. Wickham, H. *ggplot2: elegant graphics for data analysis*. (Springer, 2016).
6. Butlin, R., Collins, P. & Day, T. The effect of larval density on an inversion polymorphism in the seaweed fly, Coelopa frigida. *Heredity* **52**, 415–423 (1984).
7. Mérot, C. *et al.* Intercontinental karyotype–environment parallelism supports a role for a chromosomal inversion in local adaptation in a seaweed fly. *Proc Biol Sci* **285**, (2018).
8. Day, T., Dawe, C., Dobson, T. & Hillier, P. A chromosomal inversion polymorphism in Scandinavian populations of the seaweed fly, Coelopa frigida. *Hereditas* **99**, 135–145 (1983).
9. Hamilton, N. E. & Ferry, M. ggtern: Ternary Diagrams Using ggplot2. *Journal of Statistical Software* **87**, 1–17 (2018).
10. Gilburn, A. S., Crean, C. S. & Day, T. H. Sexual selection in natural populations of seaweed flies: variation in the offspring fitness of females carrying different inversion karyotypes. *Proc. R. Soc. Lond. B* **263**, 249–256 (1996).
