# Supplementary material for: Balancing selection via life-history trade-offs maintains an inversion polymorphism in a seaweed fly
Source: Nat Commun. 2020 Feb 3;11:670. doi: 10.1038/s41467-020-14479-7 (PMC6997199; doi:10.1038/s41467-020-14479-7)
Supplement: Supplementary file 3 — Reporting Summary [file 41467_2020_14479_MOESM3_ESM.pdf]

nature research

Corresponding author(s): Claire Mérot
Last updated by author(s): Dec 21, 2019

Reporting Summary

Nature Research wishes to improve the reproducibility of the work that we publish. This form provides structure for consistency and transparency in reporting. For further information on Nature Research policies, see Authors & Referees and the Editorial Policy Checklist .

Statistics

For all statistical analyses, confirm that the following items are present in the figure legend, table legend, main text, or Methods section.

n/a	Confirmed	
☐	☒	The exact sample size (n) for each experimental group/condition, given as a discrete number and unit of measurement
☐	☒	A statement on whether measurements were taken from distinct samples or whether the same sample was measured repeatedly
☐	☒	The statistical test(s) used AND whether they are one- or two-sided<br>*Only common tests should be described solely by name; describe more complex techniques in the Methods section.*
☐	☒	A description of all covariates tested
☐	☒	A description of any assumptions or corrections, such as tests of normality and adjustment for multiple comparisons
☐	☒	A full description of the statistical parameters including central tendency (e.g. means) or other basic estimates (e.g. regression coefficient) AND variation (e.g. standard deviation) or associated estimates of uncertainty (e.g. confidence intervals)
☐	☒	For null hypothesis testing, the test statistic (e.g. F, t, r) with confidence intervals, effect sizes, degrees of freedom and P value noted<br>*Give P values as exact values whenever suitable.*
☒	☐	For Bayesian analysis, information on the choice of priors and Markov chain Monte Carlo settings
☒	☐	For hierarchical and complex designs, identification of the appropriate level for tests and full reporting of outcomes
☒	☐	Estimates of effect sizes (e.g. Cohen's d, Pearson's r), indicating how they were calculated

Our web collection on statistics for biologists contains articles on many of the points above.

Software and code

Policy information about availability of computer code

Data collection	Simulation code is available at https://github.com/enormandeau/coelopa_fastsim with parameters files used to generate the simulated data.
Data analysis	All data were analysed using the software R 3.5.0.

For manuscripts utilizing custom algorithms or software that are central to the research but not yet described in published literature, software must be made available to editors/reviewers. We strongly encourage code deposition in a community repository (e.g. GitHub). See the Nature Research guidelines for submitting code & software for further information.

Data

Policy information about availability of data

All manuscripts must include a data availability statement. This statement should provide the following information, where applicable:

- Accession codes, unique identifiers, or web links for publicly available datasets
- A list of figures that have associated raw data
- A description of any restrictions on data availability

Experimental data and simulated data used for all figures are available as an associated source file.

Field-specific reporting

Please select the one below that is the best fit for your research. If you are not sure, read the appropriate sections before making your selection.

☐ Life sciences ☐ Behavioural & social sciences ☒ Ecological, evolutionary & environmental sciences

Ecological, evolutionary & environmental sciences study design

All studies must disclose on these points even when the disclosure is negative.

Study description	Experimental evolution in seaweed flies Coelopa frigida
Research sample	16 replicates from two populations of origins, split on different substrate and kept for 5 generation
Sampling strategy	Genotyped individuals were sampled randomly in each replicate and each generation - 24 males and 24 females.
Data collection	Data Collection was the responsability of Claire Mérot for the experiment. Simulations were performed by Eric Normandeau.
Timing and spatial scale	Original sampling of generation 0 in May 2017 - june 2017.
Data exclusions	20 replicates were started at geenration 1 but we only genotyped and analysed 16 replicates which had no crash of population below 100 adults.
Reproducibility	experiment includes several replicates
Randomization	not applicable
Blinding	individual adults or eggs chosen for genotyping were drawn randomly from a pool of samples kept in ethanol for each generation and each replicate.

Did the study involve field work? ☒ Yes ☐ No

Field work, collection and transport

Field conditions	Flies were collected at two occasions in two localities
Location	Cap Espoir, Québec (CE: 48.43087, -64.32778) Kamourasksa, Québec (KA: 47.56294, -69.87375)
Access and import/export	not applicable
Disturbance	About a thousands flies were collected randomly with insect nets from a population over tens of thousands of flies.

Reporting for specific materials, systems and methods

We require information from authors about some types of materials, experimental systems and methods used in many studies. Here, indicate whether each material, system or method listed is relevant to your study. If you are not sure if a list item applies to your research, read the appropriate section before selecting a response.

Materials & experimental systems

n/a	Involved in the study	
☒	☐	Antibodies
☒	☐	Eukaryotic cell lines
☒	☐	Palaeontology
☐	☒	Animals and other organisms
☒	☐	Human research participants
☒	☐	Clinical data

Methods

n/a	Involved in the study	
☒	☐	ChIP-seq
☒	☐	Flow cytometry
☒	☐	MRI-based neuroimaging

Animals and other organisms

Policy information about studies involving animals; ARRIVE guidelines recommended for reporting animal research

Laboratory animals	Coelopa frigida flies
Wild animals	Wild samples were collected with nets and brought back to the lab for egg laying.
Field-collected samples	All flies were raised in controlled conditions in thermo-regulated chambers (25°C).
Ethics oversight	No ethical approuval was required for invertebrates

Note that full information on the approval of the study protocol must also be provided in the manuscript.
