# Supplementary material for: Balancing selection via life-history trade-offs maintains an inversion polymorphism in a seaweed fly
Source: Nat Commun. 2020 Feb 3;11:670. doi: 10.1038/s41467-020-14479-7 (PMC6997199; doi:10.1038/s41467-020-14479-7)
Supplement: Supplementary file 2 — Peer Review File [file 41467_2020_14479_MOESM2_ESM.pdf]

Reviewers' comments:

Reviewer #1 (Remarks to the Author):

This very nice paper explores the maintenance of a chromosomal inversion polymorphism in the seaweed fly Coelopa frigida. The authors use a combination of experimental evolution and computer simulations to understand how the polymorphism is maintained. They show, through impressive experiments and exhaustive combinations of simulated parameters, that the polymorphism can be maintained under a wide range of scenarios, but most likely involving antagonistic pleiotropy between reproductive success and survival (mediated through variation in development time). The degree of environmental heterogeneity, density and sex-specific selection may all also play important roles in determining whether the polymorphism is stable and what the equilibrium frequencies of the different haplotypes are. Under many scenarios antagonistic pleiotropy leads to overdominance of fitness.
The polymorphism has been extensively studied since the early-mid 1980s, principally by Butlin and colleagues. Thus the observation that this polymorphism can be maintained by heterozygote advantage is not completely new. Where this study is really impressive though is that (i) the experiments where repeated across 4 replicates, which showed very consistent patterns and (ii) the simulations really do show how a very diverse set of scenarios (which are sensibly and experimentally parameterised) can lead to antagonistic pleiotropy and the persistence of the polymorphism. I feel for these reasons the paper is lifted beyond an incremental advance of our understanding of this particular system to something of far more general and applicable relevance to evolutionary genetic research.
I do not have any major criticisms of the work – I found it to be well written, convincing, and very carefully planned. I have a few minor comments that I hope the authors might consider when revising it, but they should be easy to address.

1) I think it would be useful to provide a bit more background to the polymorphism, especially if any genomics work has been done on it. What size is it (both in Mbp and as a proportion of the genome)? Do we know how many genes reside within it? This sort of information would help the reader evaluate some of the discussion about genetic loads etc.

2) Lines 204-207. Isn't a decreased excess of αα homozygotes inevitable as the frequency of α goes up? As the frequency of α increases, so does the expected number of homozygotes. Therefore, it becomes mathematically harder for the observed number of αα homozygotes to exceed the expected number. Negative-frequency dependence may not be required to explain the data (consistent with Table S8 / line 240).

3) It is my understanding that in the simulations, the duration of available habitat is an independent random draw for each individual. However, in the real world this independence is almost certain to be violated. For example, an exceptionally high tide that sweeps away seaweed will affect many individuals simultaneously. As I understand it, that non-independence is not built into the simulations and I wonder whether it should be? (or at least discussed).

Some minor typos / editorial suggestions.
Line 151 – replace 'origin of' with 'original'
Line 219 – replace 'male sire a disproportionate' with 'males sire a disproportionately'
Line 224 – replace 'build' with 'built'
Line 257-258 – replace 'slow-developing males to reach adulthood' with 'slow-developing males from reaching adulthood'
Line 265 – 'as observed IN many natural populations'
Line 652 – 'Genic', not 'genie'. Mistake seems to be on the Heredity webpage, but not the pdf.

Reviewer #2:

[Note from the editor] This reviewer provided all their comments confidentially to the editor; they made several suggestions which I paraphrase here:

1) The main results are rather similar to previous work, including (for example) Butlin, Day et al. Some additional context, to more fully explain how this relates to previous work and where the novelty lies, would be welcome.

2) The results are discursive and lack statistical support in the text. To clarify journal policy, we do ask that full methods and all the main results are reported in the main manuscript rather than SI.

3) The discussion is long but repetitive. The results and main implications are clear and easily summarised; the authors should talk more about selection via male success and how this affects the maintenance of the polymorphism.

Reviewer #1 (Remarks to the Author):

This very nice paper explores the maintenance of a chromosomal inversion polymorphism in the seaweed fly Coelopa frigida. The authors use a combination of experimental evolution and computer simulations to understand how the polymorphism is maintained. They show, through impressive experiments and exhaustive combinations of simulated parameters, that the polymorphism can be maintained under a wide range of scenarios, but most likely involving antagonistic pleiotropy between reproductive success and survival (mediated through variation in development time). The degree of environmental heterogeneity, density and sex-specific selection may all also play important roles in determining whether the polymorphism is stable and what the equilibrium frequencies of the different haplotypes are. Under many scenarios antagonistic pleiotropy leads to overdominance of fitness.

The polymorphism has been extensively studied since the early-mid 1980s, principally by Butlin and colleagues. Thus the observation that this polymorphism can be maintained by heterozygote advantage is not completely new. Where this study is really impressive though is that (i) the experiments where repeated across 4 replicates, which showed very consistent patterns and (ii) the simulations really do show how a very diverse set of scenarios (which are sensibly and experimentally parameterised) can lead to antagonistic pleiotropy and the persistence of the polymorphism. I feel for these reasons the paper is lifted beyond an incremental advance of our understanding of this particular system to something of far more general and applicable relevance to evolutionary genetic research.

I do not have any major criticisms of the work – I found it to be well written, convincing, and very carefully planned. I have a few minor comments that I hope the authors might consider when revising it, but they should be easy to address.

Authors' response: We thank the reviewer for his/her enthusiasm about our study and the insightful comments which we have tried to address in the revised version.

1) I think it would be useful to provide a bit more background to the polymorphism, especially if any genomics work has been done on it. What size is it (both in Mbp and as a proportion of the genome)? Do we know how many genes reside within it? This sort of information would help the reader evaluate some of the discussion about genetic loads etc.

Authors' response: Thank you for this suggestion. We added to the Introduction, on L85-87 "Here, we focused on the seaweed fly *Coelopa frigida*, whose natural populations are all polymorphic at a large chromosomal inversion on chromosome I, representing about 10% of the genome (25MB) and containing one thousand genes (26-28)."

2) Lines 204-207. Isn't a decreased excess of αα homozygotes inevitable as the frequency of α goes up? As the frequency of α increases, so does the expected number of homozygotes. Therefore, it becomes mathematically harder for the observed number of αα homozygotes to exceed the expected number. Negative-frequency dependence may not be required to explain the data (consistent with Table S8 / line 240).

Authors' response: This is true, and it is likely the reason why models without frequency-dependent effect also capture the decrease in the excess of αα homozygotes. We reformulated the results to

remove the emphasis on the experimental pattern. Therefore, in the revised manuscript, we simply mention that frequency-dependent models were also explored because the hypothesis of frequency-dependence is not aberrant in the system, a large male will likely have an individual success that is much higher when competition only occurs with smaller males. Yet, as it is not required to explain the data and does not provide a better fit than the simpler model, we chose in the subsequent analysis to focus on more simple models.

See L209-213 “Models including a negative frequency-dependent effect on mating success were also explored, to account for a possible higher male-male competition between large-sized males when the proportions of $\alpha\alpha$ increased. Although those models adequately fit the experimental data, the global fit was not significantly better compared to simpler models (Supplementary Figure 5, Supplementary Table 8).”

3) It is my understanding that in the simulations, the duration of available habitat is an independent random draw for each individual. However, in the real world this independence is almost certain to be violated. For example, an exceptionally high tide that sweeps away seaweed will affect many individuals simultaneously. As I understand it, that non-independence is not built into the simulations and I wonder whether it should be? (or at least discussed).

Authors’ response: The reviewer is right that the duration of habitat availability may affect all individuals living in the same habitat. This is modelled by the procedure that draws individual duration of wrackbed availability from the same distribution, centered on a common mean value, for all individuals of a given simulation. For instance, for a simulation with a mean duration of habitat availability (A_{mean} parameter) set to 14 days and variability (A_{var} parameter) of 2 days, individual duration of habitat will vary between 12 and 16 days. Variability reflects the fact that, in nature, not all eggs are laid at the same day, but being centered on the same mean (14 days) reflects the fact that the tide will remove the habitat at the same time for all individuals.

We apologize that this aspect was unclear in the previous manuscript. We added a few sentences to better explain it in the Methods section L484-492: “To model the duration of habitat availability, this value was randomly-drawn for each individual from a uniform distribution determined by two parameters: mean duration (A_{mean}) and variability (A_{var}). Mean duration was shared by all individuals of a given simulation, and thus takes into account the non-independence in the availability of the habitat, *i.e.* the fact that when the wrackbed is removed by a high tide or a storm, all individuals are affected at the same time. Variability represents individual heterogeneity in the total duration before the wrackbed is removed, which depends for instance, on the date at which a given egg was laid or whether all patches of wrackbed are deposited or removed at the same time.”

Some minor typos / editorial suggestions.

Authors’ response: Thanks for pointing the typos out, they have been corrected in the revised manuscript

Line 151 – replace ‘origin of’ with ‘original’
Line 219 – replace ‘male sire a disproportionate’ with ‘males sire a disproportionately’
Line 224 – replace ‘build’ with ‘built’
Line 257-258 – replace ‘slow-developing males to reach adulthood’ with ‘slow-developing males from reaching adulthood’
Line 265 – ‘as observed IN many natural populations’

Line 652 – 'Genic', not 'genie'. Mistake seems to be on the Heredity webpage, but not the pdf.

Reviewer #2:

[Note from the editor] This reviewer provided all their comments confidentially to the editor; they made several suggestions which I paraphrase here:

Authors' response: Thank you for summarizing the reviewer's remarks and please receive our apologies if we have not answered the comments adequately or not specifically enough. We have tried our best but this is a challenging task given the broadness of the reviewer's comments.

1) The main results are rather similar to previous work, including (for example) Butlin, Day et al. Some additional context, to more fully explain how this relates to previous work and where the novelty lies, would be welcome.

Authors' response: This study indeed largely builds on the previous (tremendous) amount of empirical work on *Coelopa frigida's* life-history and inversion polymorphism. We acknowledge this extensively throughout the text, and have improved this aspect in the revisions even further, by comparing our results to previous results and by quoting a total of 16 previous studies on *Coelopa frigida.* Please see in the introduction (L84-102), the results (L156-158, L169-170, L194-199) and discussion (L324-345, L357-361, L345-394).

As requested, we have also tried to explain better how our study brings novelties to previous efforts on this system and in fact, on most systems that have addressed the same fundamental question regarding the maintenance of polymorphism by means of antagonistic pleiotropy. We think that our study does bring novelty to the study of this inversion polymorphism in *Coelopa frigida* by focusing on the context of antagonistic pleiotropy and by quantifying the different components of fitness within the same study. Notably, previous estimates of survival or bias in mating success were estimated by genotyping the inversion with an allozyme marker, and scored at the early adult stage/late larval stage. Here, by using a SNP marker, we were able to genotype directly the eggs and thus to better disentangle the different components of fitness. This is not trivial because it shows for instance that heterozygote excess emerged already at the egg stage, thus results not only from higher survival of AB genotypes (which relates to genetic load for instance) but also from non-random mating. Besides, to our knowledge, this is the first time that an experiment follows the evolution of the inversion frequency in the same experimental populations of *Coelopa frigida* for several generations and with several replicates. Moreover, our analysis is complemented by extensive simulations that let us explored more systematically a wide range of (sensible) parameters. With this, we have now the power to numerically test the hypothesis that emerged previously from the literature (and from our data) but were never modelled altogether, namely that the interplay between the life-history trade-off, environmental variability and dominance lead to a protected polymorphism.

We edited the text throughout to clarify this. For instance, in L113-119 the revised version now better explains the aim of our study and these novel aspects. "First, we use experimental evolution (Figure 1B) to follow the inversion frequencies for 5 generations and to estimate the relative fitness associated with each genotype. We took advantage of being able to genotype the inversion in the eggs to specifically dissect the different components of fitness, such as egg-to-adult survival and reproductive bias. Second, realistic numerical simulations based on these experimentally-estimated

parameters allowed us to quantify the contributions of antagonistic pleiotropy in the maintenance of this inversion polymorphism."

Finally, we would like to emphasize that this study and the modelling exploration goes beyond the specific system of *Coelopa frigida,* but more generally addresses the maintenance of polymorphism through antagonistic pleiotropy. In particular, recent theoretical modelling (Brown & Kelly, 2018; Zajitschek & Conallon, 2018) have raised the importance to take into account how antagonistic pleiotropy interplays with realistic factors such as dominance, sex-specific effects and/or environmental heterogeneity in the maintenance of polymorphism. This is a matter that we address here with empirical data and numerical simulations. Therefore, although putting our results in the context of previous work on *C. frigida* polymorphism is important (and we have tried to improve that in the revision), we think that orientating the discussion on other examples of antagonistic pleiotropy or species with similar inversions polymorphism associated with life-history trade-off will be more interesting for the broad readership of Nature Communications

2) The results are discursive and lack statistical support in the text. To clarify journal policy, we do ask that full methods and all the main results are reported in the main manuscript rather than SI.

Authors' response: Some methodological information or statistical output were too condensed in the previous version (due to formatting and our willingness to keep the text as readable as possible). In the revised version, we have expanded the methods to include more detail (which were previously contained in the Supplementary Methods) and added in the main text the results and the output of statistical tests that were previously only contained in figures or in supplementary tables. Results and methods are now conforming to the Nature Communications recommendations.

See for instance in the methods:

L448-451: "Variation in allele or genotype proportions between generation 1 and 5 was analysed with a generalized linear mixed model for binomial data taking into account the identity of the replicate as a random factor and substrate or population as covariates."

L463-472:"Differences between genotypes and between sexes in survival rate or in the deviation from random mating in the eggs were tested with a linear mixed model, taking into account the identity of the replicate as a random factor, and post-hoc *t*-test corrected following Benjamini & Hochberg (68). Development time was measured at generation 5 on 757 adults as the number of days between the day of oviposition and adult emergence. Differences in development time between genotypes and sex were tested with generalized linear mixed models, based on a Poisson distribution, taking into account the identity of the replicate as a random factor. Female fecundity was assessed using 90 females by counting the number of eggs laid by each female within a 12 – 24 h period and differences in fecundity between genotypes was tested with linear models and post-hoc *t*-test corrected following Benjamini & Hochberg (68)."

L506-510: "The average nRMSE over the 6 variables ($\alpha\alpha/\alpha\beta/\beta\beta$ proportions in the eggs and the adults), and over the 30 replicates, was taken as an index of fit, with the best predicting scenarios having the smallest values. Difference of mean nRMSE between the best scenarios was tested with a t-test based on the 30 replicates, corrected following (68)"

And in the results:

L126-135: "The frequency of the *α* allele, originally at 27-36% in natural populations, increased significantly between generation 1 and 5 from 28-56% to 58-75% ($z = 10.6$, $p < 0.001$, Figure 1C, Supplementary Table 1-2). This was observed in all 16 replicates, with no significant effect of the substrate ($p = 0.27$, Supplementary Table 2). Initial differences in frequency between the two origins were lost at generation 5 (Figure 1C-F, Supplementary Table 2). The increase in *α* frequency stemmed from a sharp and significant increase of *αα* homozygotes from 4-24% to 25-57% ($z = 8.8$, $p < 0.001$, Figure 1D) and a reduction in *ββ* homozygotes from 12-48% to 0-15% between generation 1 and 5 ($z = -7.9$, $p < 0.001$, Figure 1F). Proportions of α*β* heterozygotes remained stable around 50% [36-73%] between generation 1 and 5 ($z = -1.4$, $p = 0.16$, Figure 1E)."

L145-146: "Egg-to-adult relative survival was significantly affected by genotype (df=2, F=4.7, p<0.01, Figure 2A, Table 1, Supplementary Table 4, Supplementary Figure 2)."

L176-18.:" Egg genotype proportions significantly deviated from proportions expected under random mating, *i.e.* based on the Hardy-Weinberg proportions of the previous generation (combined probabilities of chi^2 tests, p=0.003). The deviation from random mating expectation in the eggs is significantly different between genotypes (df=2, F=24.9, p<0.001; Figure 2C, Supplementary Table 6, Supplementary Figure 4)."

3) The discussion is long but repetitive. The results and main implications are clear and easily summarised; the authors should talk more about selection via male success and how this affects the maintenance of the polymorphism.

Authors' response: We have re-phrased the Discussion to shorten repetitive aspects. Following the reviewer recommendation, we removed the Conclusion section to further limit repetitions. We also ensured to include more details about male reproductive success in relation to the polymorphism.

See for instance:

L194-199" Previous experiments in *C. frigida* with two males and one female demonstrated that the largest male sires a disproportionately higher number of the progeny (37). The reasons are two-fold: smaller males lose male-male competition by being dislodged by larger males (41); and, even in single-pair experiments, smaller males are more likely to be rejected by females during mating than larger males, with *ββ* males being 30 % and 20 % less attractive than *αα* and *αβ* males, respectively (36, 39, 42)."

L299-303: "Both empirical results and simulations emphasize the importance of accounting for the reproductive phase and sexual selection. In fact, when considering viability alone, the persistence of the α haplotype, and its increase in our experiment, represents a paradox that is only resolved by considering female fecundity and the reproductive bias favouring larger males (37, 42). "

L340-348: "In nature, this translates to geographic variation in male and female sizes, as well as variation in size difference among genotypes and between the sexes (26, 36). This is non-trivial because adult size, and size difference between sexes or between competitors may modify the reproductive advantage associated with the *α* allele. For instance, experiments showed that a larger size difference between two competitor males relates to a higher success of the larger male (37). In other words, local environmental conditions are expected to also affect genotype-associated fitness values both for viability and reproductive success, thus creating spatial or temporal heterogeneity in the slope of the trade-off between survival and fertility in *C. frigida*."

REVIEWERS' COMMENTS:

Reviewer #1 (Remarks to the Author):

I enjoyed reading this manuscript the first time around and had relatively minor comments then. The three main points I had were as follows:
1) More information was needed on the inversion polymorphism and its genomic architecture. This has now been reported.
2) The observation that the excess of αα homozygotes declined as the frequency of the α allele increased was, I think, an inevitablility rather than evidence of negative-frequency dependence. The authors have acknowledged this and changed the wording as a consequence. I don't think the overall conclusions are greatly impacted by this.
3) I was concerned that the simulations treated available habitats as independent for each individual, when in fact they are correlated as e.g. wave action affects all haitats more or less simultaneously. The authors have clarified how the simulations were set up, and I am no longer concerned by this.

Overall, I think the authors have done an excellent job with the revisions, and I am satisifed that they have addressed all of the points I raised earlier. I only saw a summary of the other reviewer's comments because they were sent confidentially to the editor. Therefore I am unable to judge whether that reviewer's concerns were addressed.

Reviewer #2 (Remarks to the Author):

Thank you for the revisions - the manuscript reads very nicely now.

We thank the reviewer for their positive comments and have no more points to address.

REVIEWERS' COMMENTS:

Reviewer #1 (Remarks to the Author):

I enjoyed reading this manuscript the first time around and had relatively minor comments then. The three main points I had were as follows:
1) More information was needed on the inversion polymorphism and its genomic architecture. This has now been reported.
2) The observation that the excess of αα homozygotes declined as the frequency of the α allele increased was, I think, an inevitablility rather than evidence of negative-frequency dependence. The authors have acknowledged this and changed the wording as a consequence. I don't think the overall conclusions are greatly impacted by this.
3) I was concerned that the simulations treated available habitats as independent for each individual, when in fact they are correlated as e.g. wave action affects all haitats more or less simultaneously. The authors have clarified how the simulations were set up, and I am no longer concerned by this.

Overall, I think the authors have done an excellent job with the revisions, and I am satisifed that they have addressed all of the points I raised earlier. I only saw a summary of the other reviewer's comments because they were sent confidentially to the editor. Therefore I am unable to judge whether that reviewer's concerns were addressed.

Reviewer #2 (Remarks to the Author):

Thank you for the revisions - the manuscript reads very nicely now.
